# Programmable Metamaterials with Perforated Shell Group Supporting Versatile Information Processing

**DOI:** 10.1002/advs.202417784

**Published:** 2025-04-09

**Authors:** Xiaoyuan Ma, Ziran Wang, Weipeng Zhang, Peng Yan

**Affiliations:** ^1^ Key Laboratory of High‐efficiency and Clean Mechanical Manufacture of MOE School of Mechanical Engineering Shandong University Jinan 250061 China

**Keywords:** information processing, metamaterials, mechanical intelligence, multistable mechanisms, perforated shells

## Abstract

Mechanical metamaterials have emerged as promising tools for enabling mechanical intelligence in soft machines through interaction with the external environment. Note that most representative results in the literature focused on certain features of information processing with the designs of novel metamaterials. It remains challenging to design metamaterials with more integrated information processing capabilities toward comprehensive intelligence. In this work, a novel approach employing programmable multi‐stability of perforated shells (PS) with staggered trapezoidal voids is proposed to develop transformable, information‐processing metamaterials with high‐density information. Multi‐layer information storage, encoding, decoding, and reading are achieved by designing and arranging different types of PSs under mechanical compression or magnetic actuation. In addition, various application‐oriented functionalities, such as information encryption, mechanical computing, wave amplification, and pressure transmission, are also demonstrated by taking advantage of the stable memory and tunable stiffness distributions of metamaterials. The proposed design strategy paves the way for multifunctional, miniaturized, and scalable information mechanical metamaterials, with significant potential for soft‐material‐based intelligent devices.

## Graphical abstract

This study proposes a novel mechanical metamaterial with high‐density memory based on multi‐stable perforated shells to realize multi‐layer information encoding, storage, decoding, and reading. The metamaterial can perform more application‐oriented tasks to process information, such as encryption and dynamic behavior regulation, which hopefully enables comprehensive intelligence for soft‐material‐based adaptive robots and interactive devices.

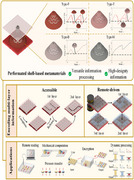

## 1 Introduction

Information processing based on the inherent properties of materials is essential for developing intelligent mechanical systems,^[^ ^1^, ^2^ ^]^ which enables the transformation of mechanical deformations to processable signals,^[^ ^3^, ^4^ ^]^ such as data storage,^[^ ^5^ ^]^ encoding,^[^ ^6^ ^]^ and computation^[^ ^7^ ^]^ without the need of electronic components. Recent advancements in fabrication technology and materials science^[^ ^8^, ^9^, ^10^, ^11^ ^]^ have greatly facilitated the development of metamaterials with progressively smaller and more complex deformable units. These metamaterials, capable of responding to multiple external stimuli,^[^ ^12^, ^13^ ^]^ have the potential to partially replace electronic devices in applications that do not require electronics.^[^ ^14^, ^15^, ^16^ ^]^

Multistable mechanisms, serving as the core components of typical information‐processing metamaterials, represent mechanical base‐*n* digits in *n* stable states.^[^ ^17^, ^18^, ^19^, ^20^ ^]^ Recent efforts has explored the use of multistable units in various forms, including 1D beams,^[^ ^21^, ^22^ ^]^ 2D lattices,^[^ ^23^, ^24^ ^]^ 3D dome shells,^[^ ^25^, ^26^, ^27^ ^]^ origami,^[^ ^28^, ^29^ ^]^ and kirigami structures,^[^ ^30^, ^31^, ^32^ ^]^ which provide a solid foundation for information storage and direct encoding within materials. Additionally, novel studies on the combinatorial strategy of nonperiodic metamaterials have decoupled the deformation between adjacent units, enabling programming at the single‐bit level.^[^ ^5^, ^33^, ^34^ ^]^ They address fundamental challenges in information processing, such as storage, transfer, encoding, and reading^[^ ^35^, ^36^ ^]^ and facilitate advanced functionalities like mechanical computing and information encryption.^[^ ^37^, ^38^ ^]^

Despite the significant progress, substantial challenges remain in achieving intelligent information processing analogous to organisms. First, a major challenge lies in constructing programmable metamaterials that can reconfigure their functionalities.^[^ ^39^, ^40^ ^]^ The single information processing function constrains the ability of metamaterials to accommodate a range of tasks.^[^ ^17^, ^41^ ^]^ Second, the challenge of balancing space‐efficient structures with high‐density information remains unsolved. Current research has improved information density at the expense of architectural complexity. For example, *n* bistable mechanisms arranged in series yield 2^*n*^ states per bit but result in bulkier, impractical systems. Through rational mechanism design rules, recent studies have proposed enabling metamaterials to perform relatively complex mechanical computational functions, such as constructing shift registers,^[^ ^42^, ^43^ ^]^ mechanical neural networks,^[^ ^28^, ^44^, ^45^ ^]^ and finite state machines.^[^ ^15^ ^]^ While these designs allow metamaterials to process more information, they significantly increase architectural complexity. Recently, harnessing bistability and scalability in dome shell has attracted growing interest for reprogrammable metamaterials.^[^ ^5^, ^25^, ^27^ ^]^ Additionally, the introduction of well‐designed holes in continuous shell enables programmable multistability,^[^ ^46^, ^47^, ^48^, ^49^, ^50^ ^]^ which has the potential to substantially improve the information density but have rarely been explored. Third, most existing studies focus on static information processing, lacking the capability to handle the dynamic scenarios such as waves and vibrations. Although recent studies have shown that stimulus‐responsive metamaterials can can program elastic wave dynamics,^[^ ^51^, ^52^ ^]^ the simultaneous processing of static and dynamic information still needs to be explored.^[^ ^53^, ^54^, ^55^ ^]^

**Figure 1:**
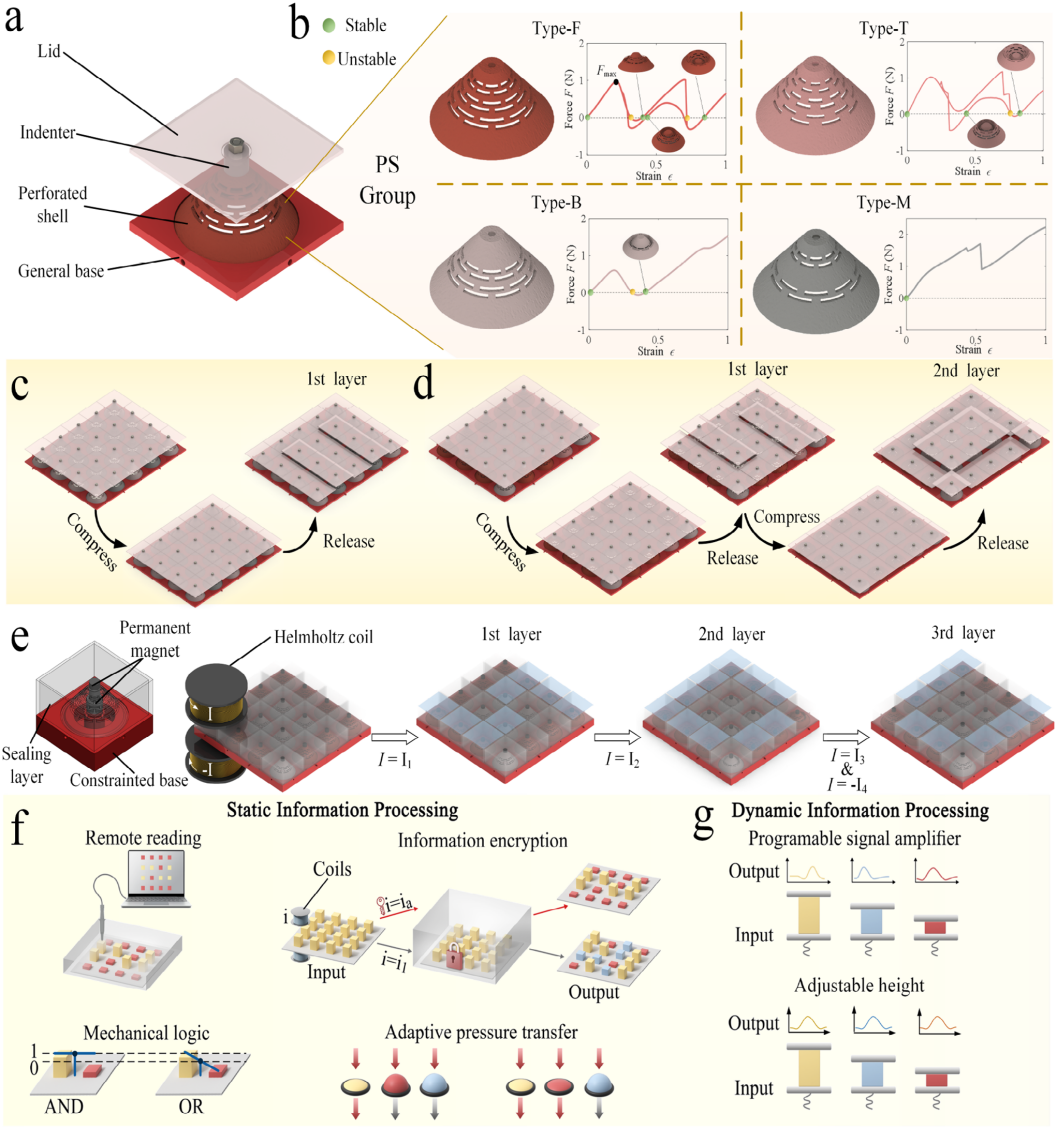
Design concept of perforated shell‐based metamaterials. a) Schematic illustration of an unit‐cell based on a perforated shell. b) Perforated shell group and their typical force‐displacement response curves. c,d) Storage and encoding of multi‐layer information by compression. e) Remote reading of multi‐layer information under magnetic actuation. f,g) The metamaterials are used in static and dynamic information processing.

In this study, we propose a design framework based on the perforated shell group (PS group, it is designed to support monostable to four stable states, **Figure** **1**a,b) to build intelligent mechanical metamaterials with high‐density information and space‐efficient structure. These metamaterials enable adjustable multistability and snap‐through/back behavior, allowing the storage and encoding of multi‐layer information, which can be decoded and read through mechanical compression (Figure 1c,d). Remote operation and direct access to stored memory are also achieved by integrating permanent magnets, even when metamaterials are encapsulated (Figure 1e). Additionally, our metamaterials perform advanced information processing functions, such as mechanical logic and data encryption, using the inherent properties of the materials (Figure 1f). Finally, we demonstrate dynamic information‐oriented applications based on metamaterials, such as tunable wave amplification and adaptive pressure transmission (Figure 1g). It is clearly demonstrated that the proposed approach has more versatile information processing capabilities, with potential applications in soft‐material‐based intelligent devices.

## 2 Results

### 2.1 Design of Perforated Shell (PS) Group

**Figure 2:**
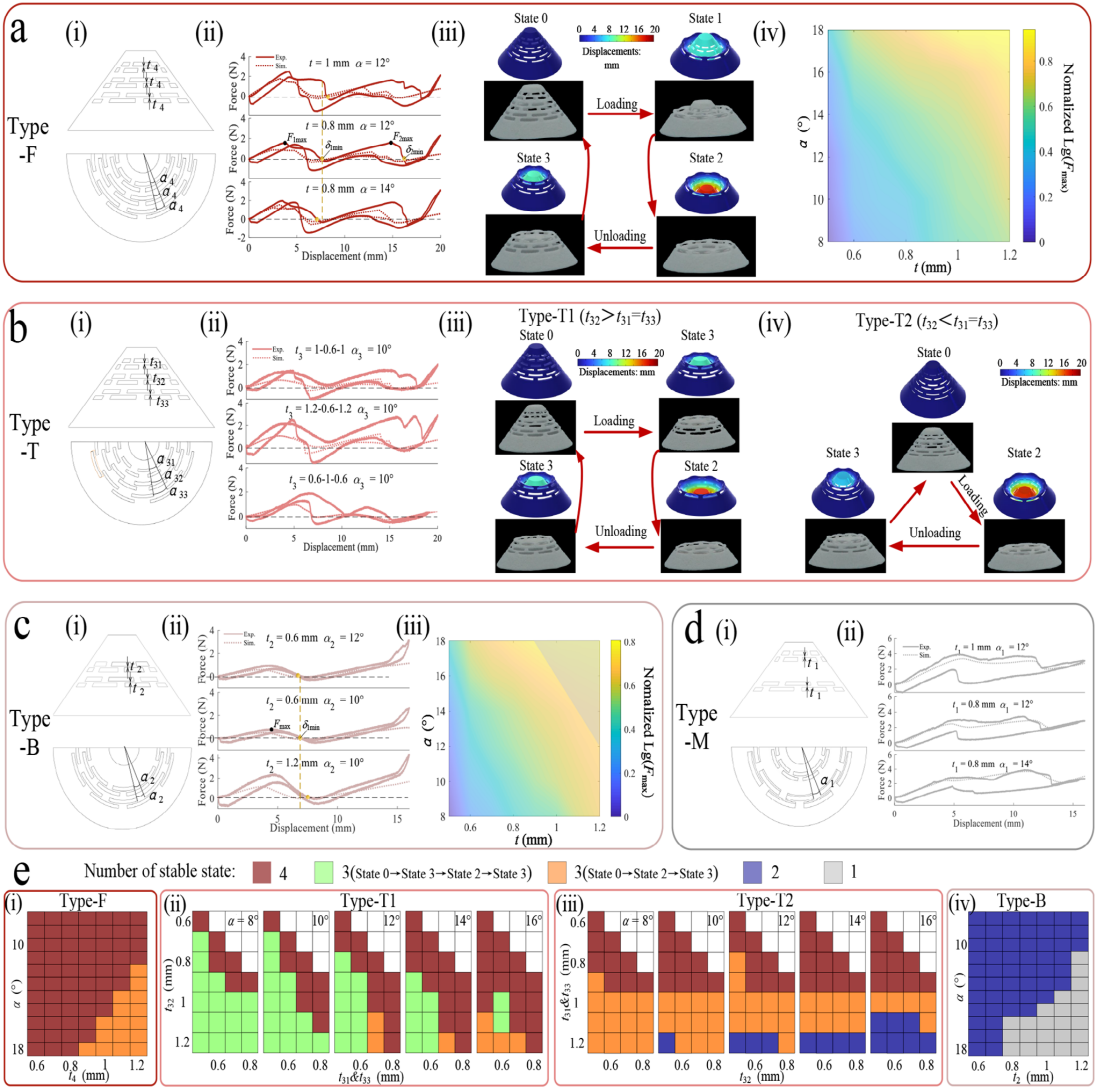
Design for four types of PS. (i) and (ii) of each subfigure (a‐d) show the design parameters of various types of PSs and their effect on the force‐displacement behavior of the structure. (a.iii), (b.iii), (c.iii) All stable configurations and simulations for Type‐F, Type‐T, and Type‐B PS. (a.iv), (c.iv) Contour of normalized snapping force of Type‐F and Type‐B,*F*_*max*_, with respect to *t* and α. e) Contour associated with the number of stable states with respect to the *t* and α, determined by FEA. i) Type‐F, ii) Type‐T1, iii) Type‐T2, iv) Type‐B.

The concept prototype called the “PS group”, includes four types of conical shells, each featuring multiple layers of staggered trapezoidal perforations (Figure 1a). These PSs exhibit varying numbers of stable states, ranging from one to four (Figure 1b; Movie S1, Supporting Information). Each unit‐cell consists of a PS, base, indenter, and lid. The PS and lid are secured with a screw‐nut assembly and the other parts are glued together (see Figure S3a, Supporting Information). The stable state of an individual PS (indicated by green dots in Figure 1b) is regarded as a discrete base‐*n* digit. When subjected to displacement perpendicular to the top surface, the PS switches to the next stable state upon reaching an unstable configuration (indicated by yellow dots in Figure 1b). Notably, once the PS transitions to the unstable configuration, it switches to the next state even after the external displacement is removed; conversely, if the displacement does not reach the threshold, the PS reverts to its initial configuration. Different types of PSs can be obtained by modifying the cutting patterns and geometric parameters of the trapezoidal holes. Specifically, they include:

- Type‐F: A PS with three rows of staggered holes, where each row has the same geometric parameters, resulting in four stable configurations (**Figure** **2**a).
- Type‐T: A PS with three rows of staggered holes, where middle row has varying parameters, leading to a tristable design (Figure 2b).
- Type‐B: A PS with no staggered holes at the apex, leading to a bistable design (Figure 2c).
- Type‐M: A PS with no staggered holes in the middle, yielding a monostable design (Figure 2d).

To guide the design of the PS, we conducted finite element analyses (FEA) and physical tests to investigate the response to variations in the projection height of adjacent layer holes *t* and circumferential angle of adjacent holes α. The remaining geometrical parameters are kept consistent across all PSs and given in Figure S2 (Supporting Information). Note that not all perforated shells satisfying the above conditions have the corresponding steady state properties. In Figure 2a–d, we select representative PSs for subsequent experiments and analyses. A detailed discussion of the stable state properties of various PSs is shown in the Figure S4 (Supporting Information).

Force‐displacement curves for Type‐F and Type‐B PSs show stable state properties across various parameters. On the other hand the parameters *t* and α regulate the minimum displacement δ_*i*min_ required for the structure to snap to the next stable state (State *i*, Figure 2a(ii), c(ii)). In addition, their snapping force *F*_*max*_ is positively correlated with their respective geometrical parameters *t* and α (Figure 2a(iv),c(iv)). The stable state configurations of Type‐F and Type‐B PS (State 0–State 3) are shown in Figure 2a(iii),c(iii), respectively, and the experiment correlates with FEA. Notably, the Type‐T PS exhibits two different force‐displacement behaviors based on the *t* of the staggered holes. Specifically, when the projected heights of the three‐row staggered perforations satisfy: *t*_32_>*t*_31_ = *t*_33_ (Figure 2b(i)), the PS reaches State 3 and then transitions to State 2 as the displacement increase, and this PS is referred to as Type‐T1 (Figure 2b(iii)). And when the projected heights of the three‐row staggered perforations satisfy: *t*_32_<*t*_31_ = *t*_33_, the PS transitions directly to State 2 while being unstable in State 1, and this PS is referred to as Type‐T2 (Figure 2b(iv)). The behavior of the above two PSs during unloading is consistent with Type‐F, both need to go through state 3 and subsequently return to the initial state. The Type‐M PS improves the snapping force by approximately 80% compared with Type‐F PS (Figure 2d(ii)). However, choosing a Type‐M PS with a larger *t* and smaller α can reduce the snapping force while maintaining its mono‐stability. The similar snapping forces of different PSs helps to reduce the stress frustration when metamaterials are compressed. Further simulations of PSs with varying parameters confirmed that these design principles are broadly applicable (Figures S5– S8, Supporting Information), thereby establishing foundation for multi‐layer information encoding and reading in PS‐based metamaterials under the control of displacement or force.

A comprehensive parametric study on the multistable behavior of all types of PS by FEA is conducted. Figure 2e shows a phase diagram associated with the number *n* of stable states with respect to the *t* and α. A Type‐F PS with a smaller *t* or α has four stable configurations. With the increase of these two parameters, the stable configuration in State 1 vanishes, whereas the others persist (Figure 2e(i)). Similarly, Type‐B PS exhibits bistable behavior when the *t* or α is small, otherwise it becomes monostable (Figure 2e(iv)). Whereas for Type‐T PS, when the α is small, the increased difference between the projection height (*t*_32_) in the middle row and projection height (*t*_31_ and *t*_33_) on both sides favors the PS to achieve our desired multistability (consistent with our definition). A small projection height difference Δ*t* (Δ*t* = |*t*_32_ − *t*_31_|) results in four stable states for the two Type‐T PSs. Finally, the combination of a large α and a significant Δ*t* induces the PS to exhibit a third multistable behavior, where the Type‐T1 PS directly transitions from State 0 to State 2, while the Type‐T2 PS is transformed into a bistable structure (Figure 2e(ii), (iii)).

### 2.2 Multi‐Layer Information Storage and Encoding Driven by Mechanical Compression

**Figure 3:**
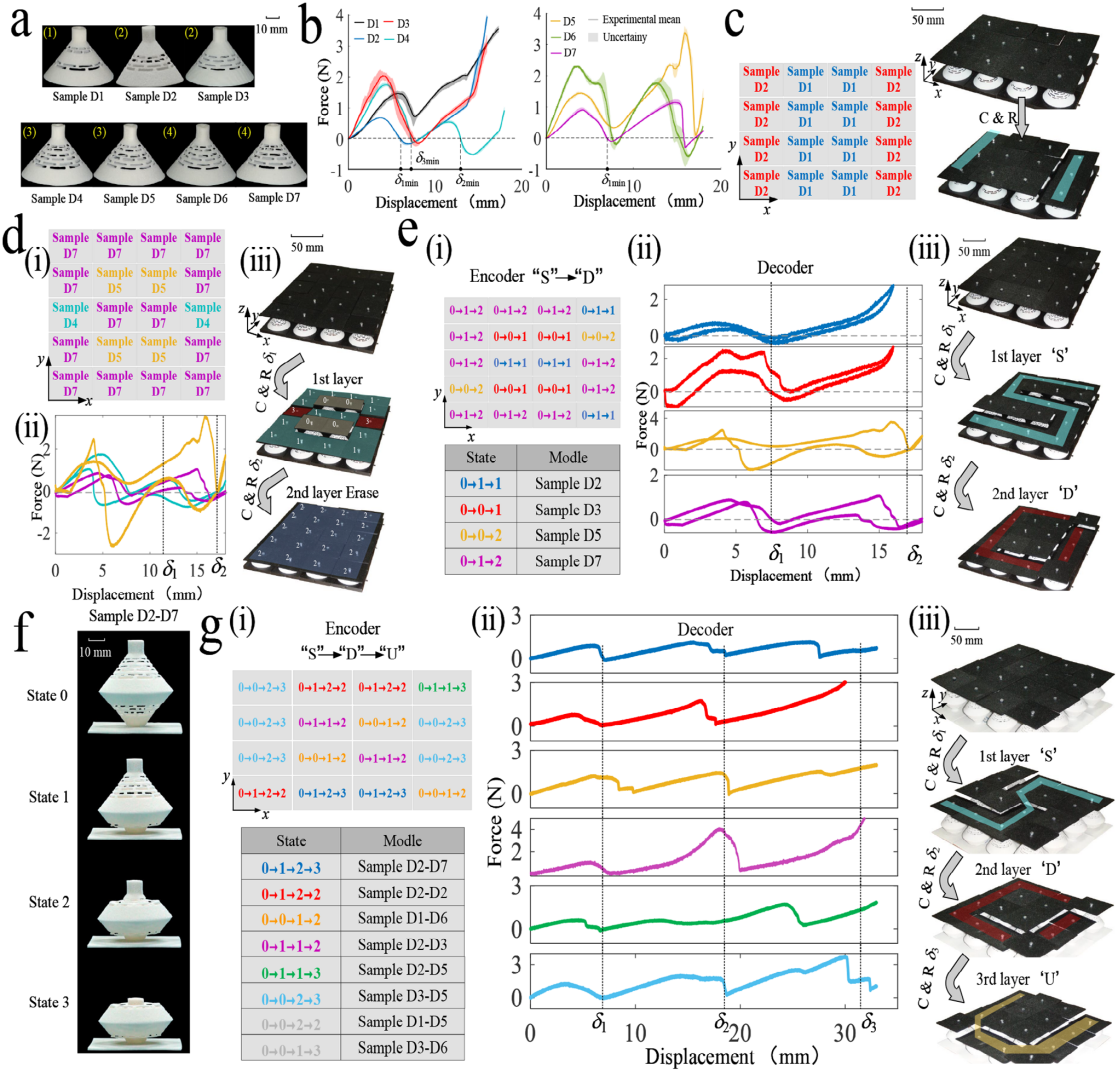
Storage, encoding, and reading of multi‐layer information in the PSs‐based metamaterial. a) Seven PS samples. Yellow numbers in parenthesis indicate the number of stable states, including the initial configuration. b) Force‐displacement curves obtained from repetitive testing of seven samples. c) Metamaterials supporting modular assembly enable one‐layer information storage. d) The first compression of the metamaterial produces the 3D information, while the second erases the information. e) Tailored two‐layer information by rationally designing the PS parameters. f) Unit structure and parameter design for realizing three‐layer information storage and reading. g) The PS‐based mechanical metamaterials are decoded to obtain three layers of tailored information in turn.

A metamaterial framework for encoding multi‐layer information is developed using seven different PSs to achieve one‐ to three‐layer information storage. The specific configurations include: Sample D1 (Type‐M, *t*_1_ = 0.6 mm, α_1_ = 8°), Sample D2 (Type‐B, *t*_2_ = 0.6 mm, α_2_ = 10°), Sample D3 (Type‐B, *t*_2_ = 1 mm, α_2_ = 10°), Sample D4 (Type‐T1, *t*_31_ = *t*_33_ = 0.6 mm, *t*_32_ = 1 mm α_3_ = 10°), Sample D5 (Type‐T2, *t*_31_ = *t*_33_ = 1.2 mm, *t*_32_ = 0.6 mm α_3_ = 10°), Sample D6 (Type‐F, *t*_4_ = 1 mm, α_4_ = 12°), and Sample D7 (Type‐F, *t*_4_ = 0.8 mm, α_4_ = 8°) as shown in **Figure** **3**a. All other geometric parameters are the same for all seven samples (Figure S2, Support Information). The repeatability testing is conducted to characterize their force‐displacement behaviors after six consecutive loading cycles. The remarkable consistency observed from testing results experimentally validates the mechanical stability and repeatability of PSs (Figure 3b), with the coefficient of variation in δ_*i*min_ less than 3% (Figure S12, Supporting Information). Additionally, the relative stability of each state can be derived as follows: State 0 > State 2 > State 1 > State 3.

Here, the metamaterials are controlled by displacement loads. The PS is selected based on the number of stable states *n* and the minimum displacement δ_*i*min_. Note that the loading speed has an insignificant impact on the force‐displacement curve of the PS (Figure S13, Supporting Information). Therefore, the transformation time of the unit‐cell is proportional to the loading speed. For illustrative purposes, all demonstrations are loaded at the speed of 1 mms^−1^. The unit‐cells are connected using an axis‐hole fit to build reconfigurable metamaterials (Figure S10, Supporting Information). And the PS is compressed and then released (C and R) to switch between different states. To illustrate the framework, Samples D1 and D2 are used for single‐layer information storage. When the applied compression displacement δ_1_ exceed δ_1min_, the external load is removed; then all Samples D2 reach State 1, while Samples D1 remain in State 0. The difference in PS heights allows the stored information to be identified (Figure 3c). Additionally, a 4 × 5 array of Samples D4, D5, and D7 is used to demonstrate information storage and erasure in the metamaterials (Figure 3d(i)). In the first step, applying a compressive displacement δ_1_ = 11 mm, followed by release, resulted in Sample D4 reaching State 3, Sample D5 returning to State 0, and Sample D7 reaching State 1. The array displays multidimensional information. In the second step, increasing the compressive displacement to δ_2_ = 17 mm and releasing it caused all PSs to transition to State 2, erasing the previously displayed information (Figure 3d(ii), (iii)).

Next, the design framework for encoding and decoding customized multi‐layer information is demonstrated. Each coding layer is independent, requiring the PSs to undergo a specific state change, as shown in Figure S10b (Supporting Information). We used the example of encoding two layers of customized information (i.e., “S”→“D”) to illustrate this framework. To this end, four PSs with different state changes are required (0→ 1 →1, 0→ 0 →1, 0→ 0 →2, 0→ 1 →2, respectively). The above four types of PSs are rationally arranged in a 4 × 5 array according to the features of the customized information. Samples D2, D3, D5, and D7 are selected to satisfy state change requirements, which can be regarded as an encoder (Figure 3e(i)). Correspondingly, the force‐displacement behaviors of the four samples can be used to decode (Figure 3e(ii)). Specifically, in the first step, Samples D2 and D7 transition to State 1 at a compressive displacement δ_1_ of 7.5 mm, while Samples D3 and D5 reverted to State 0. In the second step, all samples reached their final state when δ_2_ = 17 mm. Compression experiments confirm the ability of metamaterials to store and encode customized information sequence “S”→“D” (Figure 3e(iii) and Movie S2, Supporting Information).

Similarly, a metamaterial capable of encoding three layers of information is obtained using a unit‐cell composed of two PSs joined together. As shown in Figure 3f, the unit‐cell consisting of Samples D2 and D7 in series (D2‐D7) exhibits four stable states during loading. An example of a customized storage of three layers of information (“S”→“D”→“U”) is presented. The encoder, consisting of six unit‐cell with different state transitions (0→ 1 → 2 →3, 0→ 1 → 2 →2, 0→ 0 → 1 →2, 0→ 1 → 1 →2, 0→ 1 → 1 →3, 0→ 0 → 2 →3) is arranged as needed (Figure 3g(i)). Sample D2–D7 undergo four stable states during it is compressed. It switches from the initial state to State 1 (1st layer) when the δ_1_ is 7 mm and reaches State 2 when the δ_2_ is 18.5 mm (2nd layer), followed by State 3 when the δ_3_ is 31 mm (3rd layer). While for Sample D1–D6 (three stable states), when subject to a displacement δ_1_, the deformation of D1 dominates since the stiffness of D1 is less than that of D6, the unit‐cell reverts to the initial state after it is released (1st layer). It can not transition to State 1 (2nd layer) until the second step of displacement δ_2_ is imposed. Finally, under the compression δ_3_ of the third step, it reaches State 2 (3rd layer). The other four unit‐cells are analyzed in the same pattern, and their force‐displacement curves and snapshots of the compression process indicated that it is feasible to store three layers of information (Figure 3g(ii); Figure S10, Supporting Information). Following the above framework, 16 unit‐cells are fabricated and assembled, as shown in Figure 3g(iii). The metamaterial to encode and decode three layers of information is demonstrated as follows: first, compressing to δ_1_ and then releasing, reveals “S”; second, compressing to δ_2_ and then releasing, reveals “D”; and lastly compressing to δ_3_ and then releasing, reveals “U” (see Movie S2, Supporting Information).

### 2.3 Remote Decoding and Reading of Multi‐Layer Information Under Magnetic Actuation

**Figure 4:**
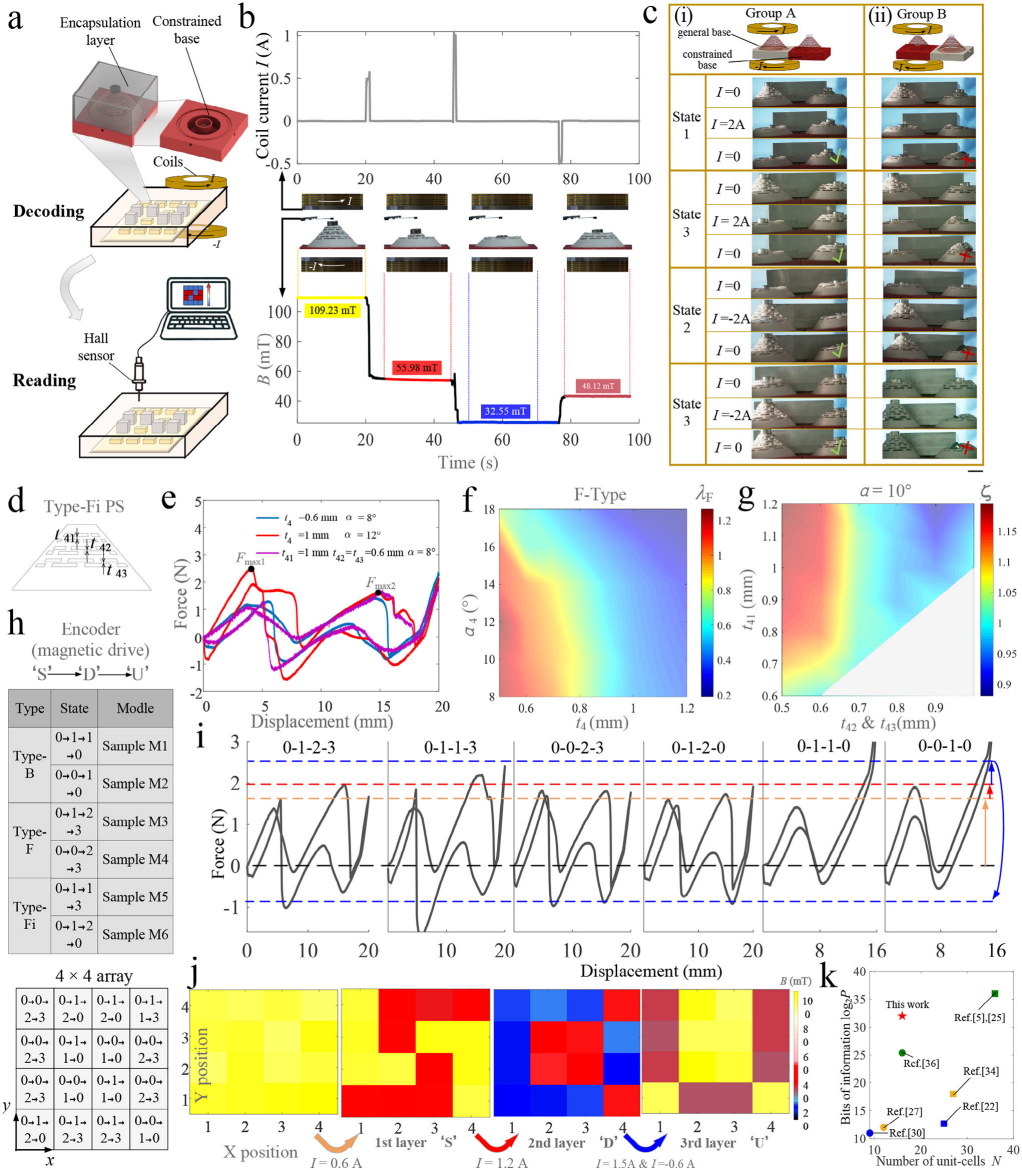
PS‐based metamaterials enable information encoding, decoding, and reading by remote magnetic actuation. a) Schematic diagram of PSs for remote magnetic actuation, decoding, and reading. b) A reversible programming cycle of the PS unit: input current in the coil (top), deformation of the PS unit (middle), and magnetic field strength in each state (bottom). c) A constrained base plate is used for the electromagnetic decoupling of adjacent unit‐cells. d) Design parameters of Type‐Fi PS. e) Comparison of force‐displacement characteristics between typical Type‐Fi PS and Type‐F PS. f) The ratio of the second snapping force *F*_*max*2_ to the first snapping force *F*_*max*1_ as a function of *t*_4_ and α_4_ in Type‐F PS. g) The ratio of λ of two types of PS with the same *t*_42_ and *t*_43_. h) Six types of PS units form a 4 × 4 array and achieve customized multi‐layer information encoding and decoding (S'', D', and U'') under magnetic actuation. i) Force‐displacement characteristics of six types of PS Units. j) The PSs‐based metamaterial decoded to obtain S, D, and U'' in sequence and displayed through a Hall module. k) Literature survey of the information density of metamaterials. Scale bars, 10 mm.

The programmable, multi‐layer metamaterial described above requires direct touches during decoding to accomplish a multi‐step operation. The information stored in the metamaterial is previously read by detecting the height differences between tessellated units, which limited its potential applications in fields like remote operations and information encryption. To address these limitations, we sandwich the top of the PS between two sets of permanent magnets (Figure 1e) and introduced a constrained base to decouple the deformation generated by the magnetic actuation of neighboring PSs (**Figure** **4**a; Figure S3b, Supporting Information). Decoding refers to the switching between different stable equilibria of all PSs in the array, achieved through magnetic interaction between two Helmholtz coils and permanent magnets (Figure 4a).

Here, two coils carry currents of equal magnitude and in opposite directions, thus producing opposite magnetic fields. The coil parameters are optimized to produce a constant driving force at the permanent magnet position (Figure 14, Supporting Information), which enhanced control over the PS deformation. The PS switches to other stable states when the magnetic force generated by the coil exceeds its snapping force. For example, we explored the state transitions of a remotely actuated PS (Sample D7): when a current of 0.5 A is applied to coils, the PS quickly switches to State 1; when a current of 1 A is applied, the PS jumps from State 1 to State 2; and when a reverse current of 0.5 A is applied, the PS switches to State 3 (Figure 4b). Once a sufficient current is applied to the coils, PS switches to a configuration between State 1 and State 2 within 80 ms (Figure S15, Supporting Information), and then reach to State 1 when currents are closed. The response time of less than 80 ms is sufficient to support various applications such as soft robots and drug release systems. The robustness of the PS to deformation paths under magnetic fields is demonstrated through 20‐cycle experiments (Movie S3, Supporting Information). There is a significant difference in the height of the PS for each state; therefore, a Hall sensor is introduced to obtain the real‐time state of the PS (below in Figure 4b). These properties allow the stored information to be decoded and directly accessed, even when the metamaterial is encapsulated.

However, the decoding of all units remains sequential rather than simultaneous. The magnetic field generated by coils affects the neighboring units owing to magnetic coupling, which could be mitigated by increasing the spacing between units. Above method inherently reduces the information density. To avoid increasing metamaterial volume, we introduce a constrained base to replace the general base in the previous unit‐cell. The diameter of the hole in the constrained base is set to 8.4 mm to accommodate a machining error of ±0.2 mm and a permanent magnet with a diameter of 8 mm, which improves the processing success rate of the constrained base and reduces the non‐axial deformation of the PS. Next, a controlled experiment is conducted to demonstrate the constrained base can limit the non‐axial deformation of the PS. For Group A, the unit‐cell with the general base is coaxial with two electromagnetic coils, and is placed to the left of the unit‐cell with the constrained base (Figure 4c(i)). As a control Group B, the positions of the two unit‐cells are reversed (Figure 4c(ii)). The left‐hand one is programmed by the magnetic field, whereas the other is affected by non‐axial magnetic forces. When a current of 2A is applied to the coils, in Group A, the unit‐cell with a constrained base is not affected by the non‐axial magnetic force, while the unit‐cell with a general base in Group B generates unanticipated deformations that persist even when the current is turned off (Figure 4c). The results show that the constrained base can suppress the non‐axial deformation of the PS in State 1 to State 3, even under high‐current excitation (*I* = 2 A). The constrained base cannot restrain the non‐axial deformation of the PS in State 0 where the permanent magnet is above the hole. However, the deformation will quickly recover once the magnetic field is removed (Movie S9, Supporting Information). Consequently, remote manipulation of metamaterials does not be affected. Of course, the excess deformation of PS in State 0 will shorten its service life.

Another noteworthy issue is that the magnetically driven PS fails to reach State 1 when the first snapping force *F*_*max*1_ of Type‐F PSs exceeds the second snapping force *F*_*max*2_ (red curve in Figure 4e). Due to the extremely short response time (less than 80 ms), the PS jumps directly to State 2 when the magnetic force up to *F*_*max*1_. Therefore, λ_F_>1 ($\lambda_{F}=\frac{F_{max2}}{F_{max1}}$, blue and purple curves in Figure 4e) is a necessary condition for the PS to reach State 1. Contour associated with the λ_F_ with respect to the *t* and α shows that the PS can reach all stable states under magnetic actuation only when the *t* and α are relatively small (Figure 4f), limiting the design space of metamaterials. To overcome the constraint, a Type‐Fi PS is introduced to regulate the ratio of the second and first snapping forces, and it is further used to construct a metamaterial framework for magnetically driven multi‐layer information storage. The design parameters of the Type‐Fi PS are chosen to satisfy the condition *t*_41_>*t*_42_ = *t*_43_ (Figure 4d). Increasing the projection height *t*_41_ significantly increases the second snapping force and has no significant effect on the first snapping force.

To show the difference between Type‐F PS and Type‐Fi PS more broadly, we also introduce a parameter ζ ($\zeta =\frac{\lambda_{\text{Fi}}}{\lambda_{F}}$, i.e., the ratio of λ of two types of PS with the same *t*_42_ and *t*_43_) that quantifies the difference in snapping force between the two types when α = 10°. As shown in Figure 4g, increasing the projection height *t*_41_ of the uppermost layer enables a broader adjustment range of the snapping force ratio, enhancing the tunability of the PS for multi‐layer information storage and decoding applications (See Figure S17, Supporting Information).

Finally, we demonstrate a complete process for encoding, decoding, and reading customized three‐layer information (“S” → “D”→ “U”) using magnetic actuation. This process employs six types of PS for magnetic actuation (referred to as Sample Mx), specifically: Sample M1 (Type‐B, *t*_2_ = 0.6 mm, α_2_ = 8°), Sample M2 (Type‐B, *t*_2_ = 0.6 mm, α_2_ = 12°), Sample M3 (Type‐F, *t*_4_ = 0.6 mm, α_4_ = 10°), Sample M4 (Type‐F, *t*_4_ = 0.8 mm, α_4_ = 12°), Sample M5 (Type‐Fi, *t*_42_ = *t*_43_ = 0.6 mm, *t*_41_ = 1 mm, α_4_ = 10°) and Sample M6 (Type‐Fi, *t*_42_ = *t*_43_ = 0.6 mm, *t*_41_ = 0.8 mm, α_4_ = 14°). The encoder used to encode the target information into the metamaterial is shown in Figure 4h. The force‐displacement behaviors of six PSs confirmed the validity of our design framework and provided a decoding scheme for each layer of information, as shown in Figure 4i. Decoding of the three‐layer information is achieved sequentially by applying magnetic forces corresponding of to the three colored dotted lines in sequence (orange→red→blue). These magnetic level lines served as reference points for correct decoding; however, any force value within the range between adjacent dotted lines was sufficient for successful decoding. We demonstrate the results of decoding three layers of information (“S” → “D”→ “U”) in a 4 × 4 array (Movie S4, Supporting Information). The array is placed on an X‐Y platform and each unit‐cell is programmed sequentially using two electromagnetic coils. Each layer of information can be read using a Hall sensor module when the array encapsulated (Figure 4j; Movie S5, Supporting Information). The proposed magnetic actuation strategy further increases the information density of metamaterials. A literature review shows that this work processes more information bits (*P*) with fewer unit‐cells than existing cm‐scale metamaterials (Figure 4k).

### 2.4 Information Encryption and Logical Computing

**Figure 5:**
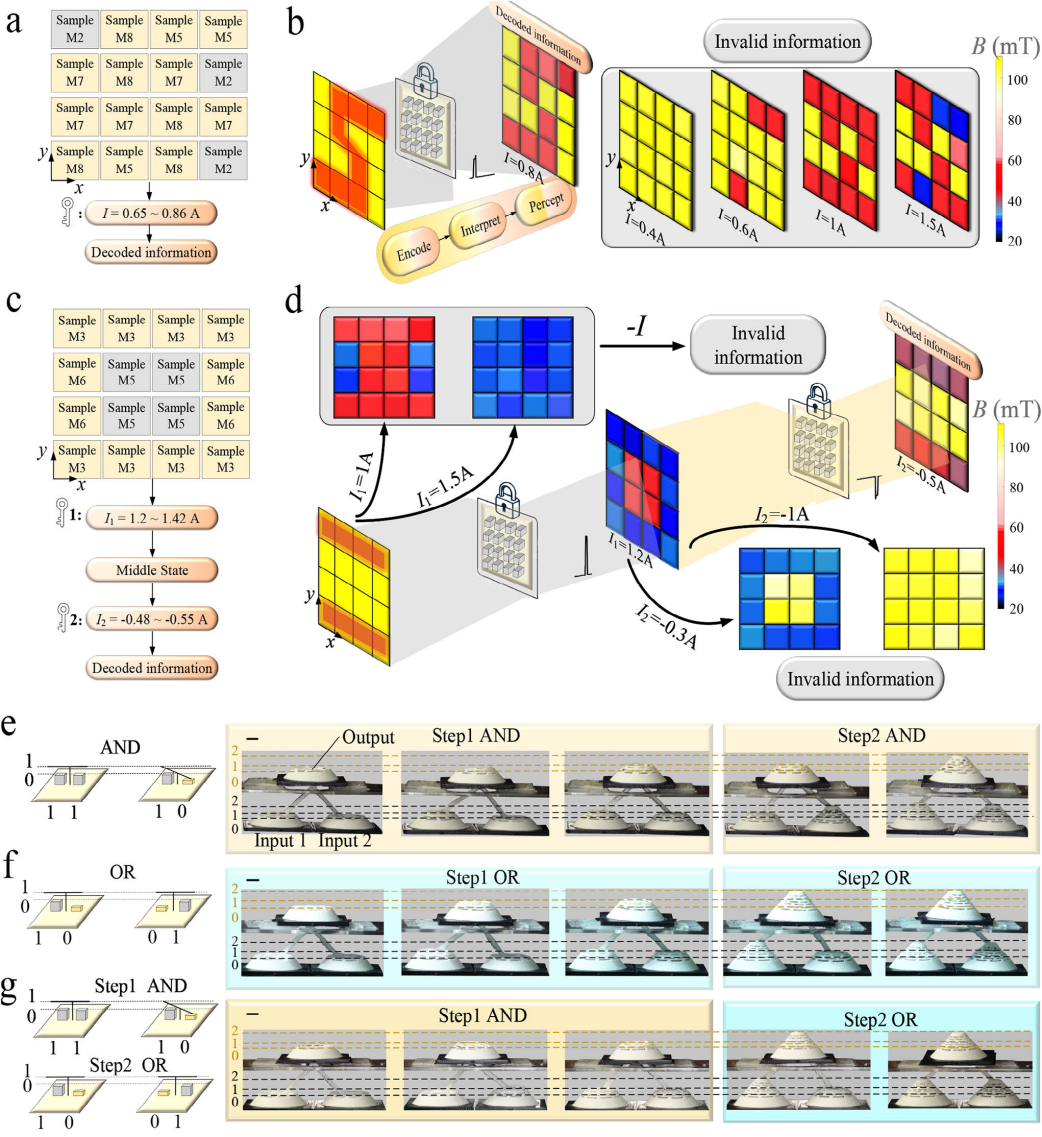
Applications of the PSs‐based metamaterial for information encryption and mechanical computing. a) Array configuration for single‐layer encrypted information “S”. b) Encrypting using a single encryption key (current magnitude) and exhibits invalid information when excited by a non‐key. c) Array configuration for dual‐key encryption information “=”. d) Using dual‐key encryption, the decoded message is output only if both input currents are correct, otherwise an invalid message is output. e–g) The PS unit is used to execute multi‐step mechanical logic: e) two‐step AND gate, f) two‐step OR gate, g)Tunable mechanical logic gates based on PSs. Scale bars, 10 mm.

The mechanical metamaterials for information encryption,^[^ ^38^ ^]^ as opposite to microwave/optical metamaterieals, can be used in special applications where electronics are prone to failure or the light cannot access.^[^ ^30^ ^]^ Existing material‐based encryption strategies typically require a trade‐off between information security and simple structure. Here, we employ the multistable PS and remote drive strategy to demonstrate a novel encryption metamaterial supporting high‐security communication without increasing structural complexity.^[^ ^21^, ^52^, ^56^ ^]^ Under the proposed design framework, the current input to the electromagnetic coils represents the “write” process, and the information detected by the Hall sensor constitutes the “read”. Notably, all unit cells are subjected to the same magnetic field, implying that the current input remained uniform. First, the information to be encrypted (letters or symbols) is pixelated into a 4×4 array, which is only displayed correctly upon entering the correct password. Second, a specific range of input currents is determined as the password for unlocking, with an obfuscation unit (gray square in **Figure** **5**a) introduced to ensure password uniqueness. Valid information can not be obtained when the input is not within the password range. Third, the encapsulated array is decrypted by sequentially applying the same magnetic field to the unit‐cells. Essentially, the encrypted information is represented by two states. When incorrect decryption causes a mismatch in these states, nonsensical symbols are displayed, signifying decryption failure. For example, to encrypt the information “S”, Samples M2, M5, M7 (Type‐M, *t*_1_ = 0.8 mm, α_1_ = 8°) and M8 (Type‐B, *t*_2_ = 0.8 mm, α_2_ = 8°) are arranged as shown in Figure 5a. The metamaterial can display the encrypted information “S” only when the coil current is within the password; otherwise, it outputs an invalid information (Figure 5b; Movie S6, Supporting Information).

A dual‐key encryption strategy is developed to enhance security without additional structures based on multistable PSs. Specifically, the encrypted information is the combination of PSs in State 3 and can be decrypted via two‐step opposite input current (i.e., a positive current followed by a reverse current). This implies that only the correct combination of forward and reverse currents can decrypt the correct target information. For example, the decryption process of a double‐layer encrypted message “=” is demonstrated. Samples M3, M5, and M6 are used to construct a double‐layer encrypted metamaterial (Figure 5c). The metamaterial displays “□” as an intermediate state when the first step current *I*_1_ is in the password range. Then, the correct reverse current *I*_2_ is applied to unlock the encrypted information, “=” (Figure 5d). The metamaterial output error information when incorrect passwords or sequences are used at any stage.

In addition, we construct multi‐step mechanical logic gates to demonstrate the broader information‐processing capabilities of PSs. The logic gate consists of three PSs configured with two inputs and one output. The top position of the PS determines the values of the inputs and outputs, with the initial state set to State 2 (corresponding to 0 for input or output). By adjusting the geometrical parameters and types of PSs, the energy released during state switching can be controlled, enabling the construction of various logic gates with different functions. For example, we take as input the two Type‐F PSs with the same parameters (*t*_4_ = 0.8 mm, α_4_ = 12°) and, as output, the top midpoint of the Type‐F PS with different parameters. The properties of the logic gates can be switched by changing the PS at the output. We construct a two‐step AND gate using PSs with larger α_4_ as output (*t*_4_ = 0.8 mm, α_4_ = 16°) as shown in Figure 5e. Conversely, a PS with a smaller *t*_4_ replacing the previous output yielded a two‐step OR gate (Figure 5f). Additionally, unlike the Type‐F PS, the relative magnitudes of the two energies required to reach the two steady states during unloading are adjustable in the Type‐Fi PS, which indicates that building two‐step logic gates with different functions is possible. By using a Type‐Fi PS (*t*_41_ = 1 mm, *t*_42_ = *t*_43_ = 0.8 mm, α_4_ = 12°) to replace the previous input again, we create a functional logic unit combining an AND gate in the first step and an OR gate in the second step (Figure 5g; Movie S7, Supporting Information). Therefore, PS provides reprogrammable capability for mechanical logic gates, which allows their operation and application in different working environments.

### 2.5 Dynamic Information Processing

**Figure 6:**
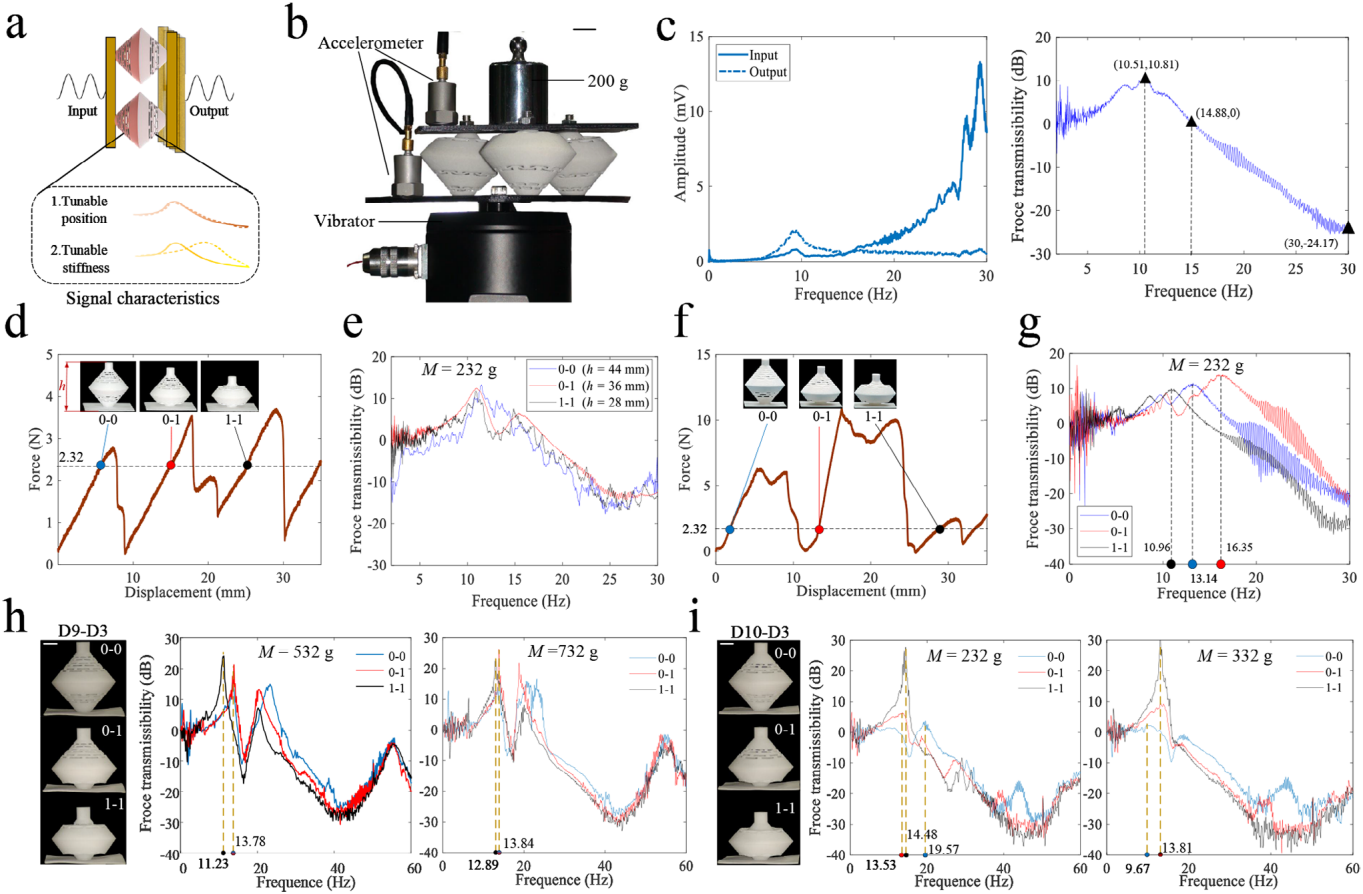
Application of PSs in processing dynamic information. a) Schematic illustration of metamaterials with multi‐modal force transfer properties. b) Experimental setup used to measure the acceleration transmissibility. c) Acceleration transmissibility under various vibration frequencies from 1 to 30 Hz. d,e) Principles and experimental results of materials for regulating height‐independent transfer properties. f,g) Principles and experimental results of materials for regulating the transfer properties of height influence. h,i) The dynamic information processing performance of metamaterials under a wider range of working conditions. Scale bars, 10 mm.

To explore another potential application, we investigated PS‐based metamaterials for amplifying or attenuating dynamic signals at specific frequencies. Here, two PSs are connected in series as a unit‐cell (Figure 3e). By adjusting the PS parameters, the system can achieve two distinct functions: modifying structural height without altering the signal transmission characteristics or adjusting the frequency range of amplified signals by changing the height (**Figure** **6**a), which arises because the compressive stiffness of the PSs either remains constant or changes after switching states. Experiments are conducted to verify the signaling characteristics on three samples with the same parameters (Sample D6‐D1) under a total load of 232 g (200 g weight and 32 g accelerometer), as shown in (Figure 6b). All experiments are performed at vibrations below 30 Hz. The results show that the material amplifies input information from 0–14.88 Hz and peaks at 10.51 Hz (Figure 6c). These findings demonstrate the potential of PS for dynamic information processing.

The force‐displacement tests of the three unit‐cells (Sample D2‐D7) show their similar stiffness in the three stable states (0‐0, 0‐1, and 1‐1), implying consistent transmission characteristics (Figure 6d). Vibration experiments confirmed our conjecture that the three states have similar frequency peaks (Figure 6e), making PS‐based metamaterials ideal for automotive seating applications. To demonstrate the reprogrammable signal processing properties of metamaterials, we introduced Sample D8 (Type‐F, *t*_4_ = 1.2 mm, α_4_ = 12°), Sample D9 (Type‐F, *t*_4_ = 1.2 mm, α_4_ = 10° and Sample D10 (Type‐B, *t*_2_ = 0.8 mm, α_4_ = 10°. Force‐displacement curves for three PS combinations (Sample D8‐D3) indicate significant stiffness variations under State 0‐0, 0‐1, and 1‐1 (Figure 6f). Experimental results on the signal transmission characteristics also show that the three states possess three different frequency peaks (Figure 6g). The input vibration signal can be amplified or attenuated within a specific range and effectively adjusted by switching the PS state, which can be used in automotive suspensions to switch between the responsive and smooth modes. By optimizing the geometrical parameters affecting PS compressive stiffness, metamaterials can amplify signals across arbitrary bandwidths (Figures S24 and S25, Supporting Information).

Furthermore, we test a boarder range of working conditions, in terms of larger loads and frequencies, to provide a more comprehensive reference for practical applications. A metamaterial consisting of four D9‐D3 samples is used to test the force transmission properties under larger loads. When all the unit‐cells are in State 0‐0 or 0‐1, the force transmission properties of the metamaterial remain almost constant when loads of 532 and 732 g are applied, respectively. However, when all the unit‐cells are switched to State 1‐1, it demonstrates different properties at vibration frequencies below 30Hz (Figure 6h). Specifically, the latter configuration shows peaks that correspond to lower frequencies. This implies that the metamaterial maintains its intended function under a maximum load of 732 g when lower input frequencies are applied. Under the same experimental configuration, state transition of the unit‐cell exhibits little effect on its force transmission properties when vibration frequencies is between 30 and 60 Hz. In particular, high‐frequency vibration results in attenuation of the output signal at all states. Moreover, tests under other loads exhibit the same results (Figure 6i). These results show that, when the input frequency is higher, metamaterials can only be used for applications that require height adjustment without changing their dynamic characteristics.

### 2.6 Adaptive Pressure Transfer

**Figure 7:**
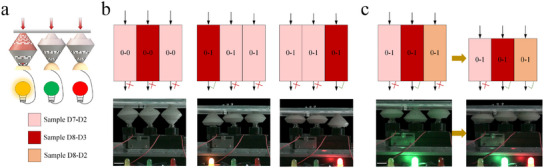
PS‐based metamaterial enables adaptive pressure transfer. a) Demonstration of a schematic of an experiment to control the pressure distribution of materials. b) Metamaterials for force transfer at specified positions. c) Metamaterials for time‐sequential transfer of pressure. Scale bars, 20 mm.

Finally, in order to demonstrate the broader applications of metamaterials, we explore the use of PSs as adaptive pressure transfer materials that allows forces to be transferred along customized paths or time sequences. For this purpose, Samples D7‐D2, D8‐D3, and D8‐D2 are used to conduct experiments. **Figure** **7**a shows the concept of a force delivery material using mechanical switches connected to LEDs of different colors. When force is applied to the top of the rigid PSs (Sample D8‐D3), it is exclusively transferred to the bottom switch, activating the matching light‐emitting diode (LED). The energy generated by the force acting on the softer (Sample D7‐D2) PSs is used to support the state switching of the completed PS, preventing sufficient force from reaching the bottom and thereby not activating the LED (Figure 7b). We also demonstrate that the pressure transfer can be sequentially controlled by introducing PSs with minor stiffness differences (Sample D8‐D3 and Sample D8‐D2). As shown in (Figure 7c, including a buffer PS (Sample D2) delayed the transmission of the pressure driven by the deformation of the PSs, resulting in sequential pressure delivery and subsequent operation of the LEDs. When the PSs are compressed, the middle LED is activated due to the high stiffness of Sample D8‐D3 in the middle position and the jump of the PSs on both sides to the next stable state. As the compressive strain increases, Sample D8‐D2 arrives at the next stable state with significant stiffness, which activated the suitable LED (Movie S8, Supporting Information).

## 3 Conclusion

In this study, we propose a novel metamaterial framework capable of multi‐layer information storage, encoding, and reading by arranging perforated shells with different multistability properties as pixels. The proposed approach allows metamaterials to process more information in smaller volumes and offer a broader design space. More importantly, we demonstrated the versatility of these metamaterials through applications in information encryption, mechanical computation, and wave amplification, as well as adaptive pressure transfer, highlighting more mechanical intelligence with the proposed method. Notably, the proposed metamaterials exhibit several features, including the ability to process both static and dynamic information, support non‐contact operation after the metamaterials are sealed, adapt to an extensive range of metamaterial frameworks for more complex information storage, and offer satisfactory lifetimes (Movie S11, Supporting Information). However, perforations in the shell will affect load‐bearing capacity and stability (Figure S18, Supporting Information), limiting reliable data storage in high vibration environments. In future research, we will investigate potential designs (e.g., self‐locking structure) to enhance the stability for a more layered scenario.

Owing to the scale‐independence of geometric nonlinearity, the proposed framework is well‐suited for miniaturized designs, making it applicable to large‐scale information manipulation. In addition, we also observe that Type‐F and ‐T1 PS exhibit additional bended steady‐state (Figure S19 and Movie S10, Supporting Information), implying that more deformation modes can be achieved by combining multiple PSs. Overall, this approach expands the toolkit for metamaterial applications and also improves the adaptability and efficiency of reprogrammable metamaterials. These unique capabilities simplify complex electronic control systems, paving the way for material‐based intelligence in platforms such as soft robotics and flexible sensors.

## 4 Experimental Section

### Fabrication of PSs and PSs‐Based Metamaterials

In the present study, for individual PSs, the additive manufacturing method of laser sintered thermoplastic urethanes (TPU) powder was utilized to obtain all perforated shells. All CAD models were imported into Cura in order to perform slicing with a layer height of 0.1 mm. For the combined PS, holes were made in the top and bottom surfaces of the model during the design to facilitate the cleaning of the powder left over from the additive manufacturing process. The base plate and lid of the unit‐cell were manufactured using the fused deposition method. For mechanically driven units, the PS was glued to the other components. For magnetically driven units, the PS was glued to the base plate and sandwiched between two sets of permanent magnets. The base plate assembled multiple unit‐cells into a metamaterial through a shaft‐hole fit.

### Mechanical Test of the PSs

The top of the PS and the connector were tightly connected by glue. The connector was fixed to the top of the PS and loading lever. The PS was clamped, and the top connector was attached the slider with a steady vertical displacement. For the combined PS, it was difficult to mechanically test it using the aforementioned method because it was perforated on both sides of the top surface. This issue was addressed by introducing a loading plate, which was securely fastened to the combined PS using a bolt‐nut fit, with the force transducer directly connected to the loading plate. Above experiments were loaded by a rail with stepper motor at a rate of 1mms^−1^ and the reaction forces were measured by force sensor (DYLY108, 10 N, Dayang Inc.) at a rate of 10000 Hz. The data were collected through a dynamic data collection device (NI 6341 DAQ card and LabVIEW program, National Instruments Inc.).

### Magnetic Actuation of the PS‐Based Metamaterials

The applied magnetic field used to drive the individual PSs was generated through two energized coils. Each coil had a resistance of about 40 ohms. The current for the coil was generated by a high power programmable power supply (TDP62300‐0, Toudapu Inc.). Experimental measurements of the generated magnetic field were performed using a Teslameter (Teslameter PMST‐1, PLARZ Inc.). To prevent the coil from overheating, the maximum applied current was set to 2A. The metamaterial was fixed to the X‐Y motion stage (RXP45XY‐H1, QRXQ Inc.) to ensure that two coils could traverse all the unit‐cells (that is, the unit‐cell were coaxial with two coils). When the unit‐cells were aligned coaxially with the coil, the X‐Y stage pauses for 3 s, during which the coils were supplied with current for 0.5 s. The current applied by the coil to each unit‐cell was consistent until all the unit‐cells were driven. This configuration allowed unit‐cells with certain parameters to be switched, while the remaining units remain unchanged.

### Experimental Setup for Remote Operation of Metamaterials

To enable remote reading of information, using the same motion stage used earlier in this study (RXP45XY‐H1, QRXQ Inc.), the magnetic field magnitude was measured using an linear Hall module (Yunfan Inc.) placed at *z* = 2 mm above each PSs. The signals collected by the Hall module were transferred to the computer via a data acquisition card (USB‐6008, National Instruments Inc.), after which the data output was realized by a program prepared in LabVIEW. Because of the large gap between the signals acquired in different states of PSs, it was possible to read the information remotely even after the metamaterial was encapsulated.

### Experimental Setups for Dynamic Information Processing

As for the application of dynamic information processing, vibration tests of PS‐based metamaterials were conducted. A phase sensitive multimeter (PSM1700 PsimetriQ, Newtons4th Ltd) generated the harmonic signals to the shaker (SA‐JZ002, Shiao Technology Inc.) to simulate vibrations. Two acceleration sensors (LC0101 from Lance Technologies Inc.) were attached to the bottom and top surfaces to record input and output accelerations. Each set of experiments was tested using three units, whose apexes were bolted to the top plate and bottoms were secured to shaker plate by glue. Two voltage regulators were connected to the acceleration sensor to power the latter. In order to obtain vibration signals at different frequencies, 1‐30 Hz signals were generated for frequency sweeping by setting up a signal source. The data were collected through a dynamic data collection device (National Instruments NI 6341 DAQ card and LabVIEW program). As for the adaptive pressure transfer experiment, three LED were connected to three switches in parallel to a DC power supply, three PSs were fixed to the top of the switches, and compressive displacement constraints were applied to the PSs using a Z‐direction motion stage.

### Finite Element Analysesg

Using SolidWorks 2018 and COMSOL Muliphysics 6.1, finite element models of individual PSs were created to perform parametric design. According to the data obtained from uniaxial tension experiments, a linear elastic material model with Young's modulus of 46.8 Mpa and Poisson's ratio of 0.45 was built. The deformation of the PS under the apex force was axisymmetric, therefore only half of the PS need to be calculated. The focus was on the *t* of the PSs (within the range 0.6 to 1.2 mm, with increments of 0.1 mm) and the α (8° to 18° increments of 1°). To improve model convergence, dividing the grid ensure at least four elements through thickness and using the displacement control strategy to simulate the behavior of model buckle. Moreover, selected the “including geometric nonlinearities” option and a global damping value of 0.0001 was selected before calculating. The model mesh density of hinges was the largest because of its large deformation, while other regions were relatively less dense in order to improve computational efficiency.

## Conflict of Interest

The authors declare no conflict of interest.

## Supporting information

Supporting Information

Supplemental Movie S1

Supplemental Movie S2

Supplemental Movie S3

Supplemental Movie S4

Supplemental Movie S5

Supplemental Movie S6

Supplemental Movie S7

Supplemental Movie S8

Supplemental Movie S9

Supplemental Movie S10

Supplemental Movie S11

## Data Availability

The data that support the findings of this study are available from the corresponding author upon reasonable request.
