# Supplementary material for: Programmable Metamaterials with Perforated Shell Group Supporting Versatile Information Processing
Source: Adv Sci (Weinh). 2025 Apr 9;12(23):2417784. doi: 10.1002/advs.202417784 (PMC12199329; doi:10.1002/advs.202417784)
Supplement: Supplementary file 1 — Supporting Information [file ADVS-12-2417784-s007.pdf]

ADVANCED SCIENCE
Open Access

Supporting Information

for *Adv. Sci.*, DOI 10.1002/advs.202417784

Programmable Metamaterials with Perforated Shell Group Supporting Versatile Information Processing

*Xiaoyuan Ma, Ziran Wang, Weipeng Zhang and Peng Yan**

Supporting Information for

Programmable metamaterials with perforated shell group supporting versatile information processing

Xiaoyuan Ma, Ziran Wang, Weipeng Zhang, Peng Yan*

Key Laboratory of High-efficiency and Clean Mechanical Manufacture of MOE, School of Mechanical Engineering, Shandong University, Jinan 250061, China

*Corresponding author. E-mail: yanpeng@sdu.edu.cn

This Supporting Information document includes:

1. Supplemental notes (SI.1—SI.17)

2. Supplemental figures (Figure S1—Figure S25)

3. Captions for Movies

Other Supporting Information for this manuscript includes the following:

Movies S1 to S11 (.mp4)

Contents

SI.1 Characterizations of materials 1

SI.2 Motif design for perforated shell groups 2

SI.3 Assembly of unit-cell 3

SI.4 Force-displacement behavior of 3D printed PS 4

SI.5 FE simulation of four kinds of PSs 5

SI.6 Influence of geometrical parameters on various types of perforated shells 8

SI.7 Mechanical compression-driven metamaterial construction principles for encoding multilayer information 10

SI.8 Cyclic repeatability testing of representative PSs 13

SI.9 Impact of loading speed on four PSs 14

SI.10 Magnetic field generated by the electromagnetic coils 15

SI.11 Parametric design of perforated shells for magnetic field actuation 18

SI.12 Mechanical stability of different types of perforated shells 20

SI.13 Constrained base design for electromagnetic actuation decoupling 22

SI.14 Principle of realizing information encryption in metamaterials 23

SI.15 Multi-step mechanical logic gate construction principle 25

SI.16 Influence of geometrical parameters of series-connected PS units on compressive stiffness 28

SI.17 Dynamic information transfer in customized frequency bands 30

Captions for Movies 33

SI.1 Characterizations of materials

To measure the stress-strain curves of TPU Nylon powder Uniaxial tensile tests were performed to characterize the elastomers. Two standard dog-bone specimens (Figure S1a) were fabricated using TPU Nylon powder and both ends of the specimens were fixed using special clamps and stretched along the horizontal direction under rail loading. Their stress was measured using a force sensor (LSB205, FUTEK Inc.). To evaluate the material parameters, the material was assumed to be completely incompressible.

The tensile tests of TPU Nylon materials are shown in Figure S1b. When the strain is small, its stress-strain curve shows good linear relationship. In this study, we consider it as a linear elastic material. According to the uniaxial tensile test results, its Young's modulus was set to 46.8 MPa. The specific material intrinsic parameters are shown in Table S1.

Table S1: Key characterization parameters for TPU Nylon materials

Properties	Young's modulus	Poisson's ratio	Density
Parameters	46.8 MPa	0.45	1100 kg/m^3

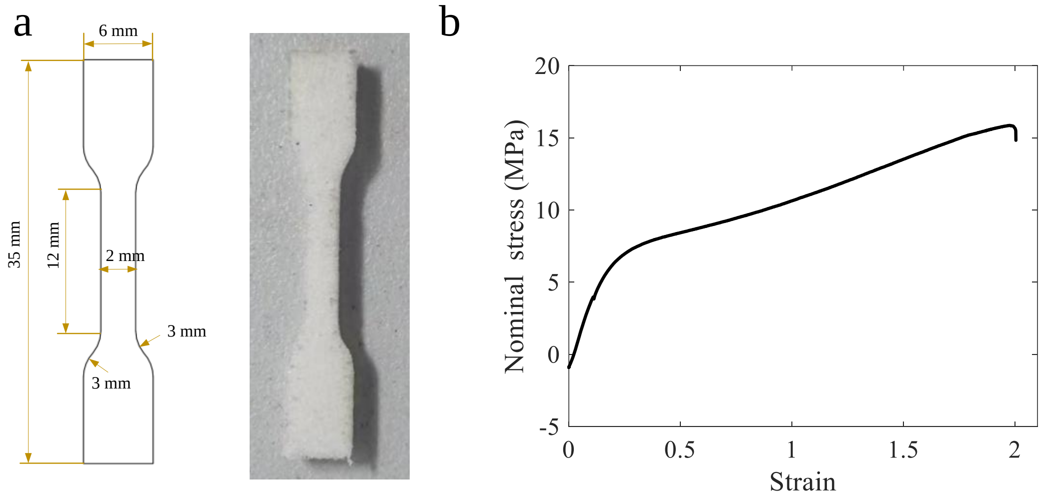

Figure S1. Measured uniaxial nominal stress-strain curves of TPU Nylon powder.

a). Dog-bone shaped plate specimen. b). The corresponding stress-strain curves.

SI.2 Motif design for perforated shell groups

In this study, all types of unit-cells of multistable perforated shell group are designed by adding perforations through deep dome shells (Figure S2a). The initial dome shell was 10 mm of the top base, 50 mm of the bottom base, and 23 mm of high. There are only two geometric parameters for all perforated shells, i.e., the distance t between perpendicularly oriented holes in the same row and the radial angle of neighboring holes α (Figure 2 in the manuscript). The Type-F PS has three layers of holes, each layer including two lines of staggered holes (Figure S2b). The height h of the holes in each layer is 3 mm, the spacing l between adjacent layers is 4.5 mm and the distance c of the bottom hole from the bottom edge is 8 mm. Based on the graphical design of the Type-F PS, the Type-T PS was constructed by making the 3 sets of parameters t different (Figure S2c). Then the size relation of the three sets of t of the PS ((i.e., Type-T1: t_1=t_3 < t_2; Type-T2: t_1=t_3 > t_2)) was varied to bifurcate the Type-T into two PS's with different stable state properties. Type-B PS with bistable properties was obtained by retaining two layers of staggered holes at the bottom (Figure S2d). The parameter settings are consistent with the previous PS. The monostable Type-M PS was obtained by retaining the staggered holes in the first and third layers (Figure S2e). Due to the presence of holes, Type-M, despite being monostable, has a buckling force close to that of other types of perforated shells.

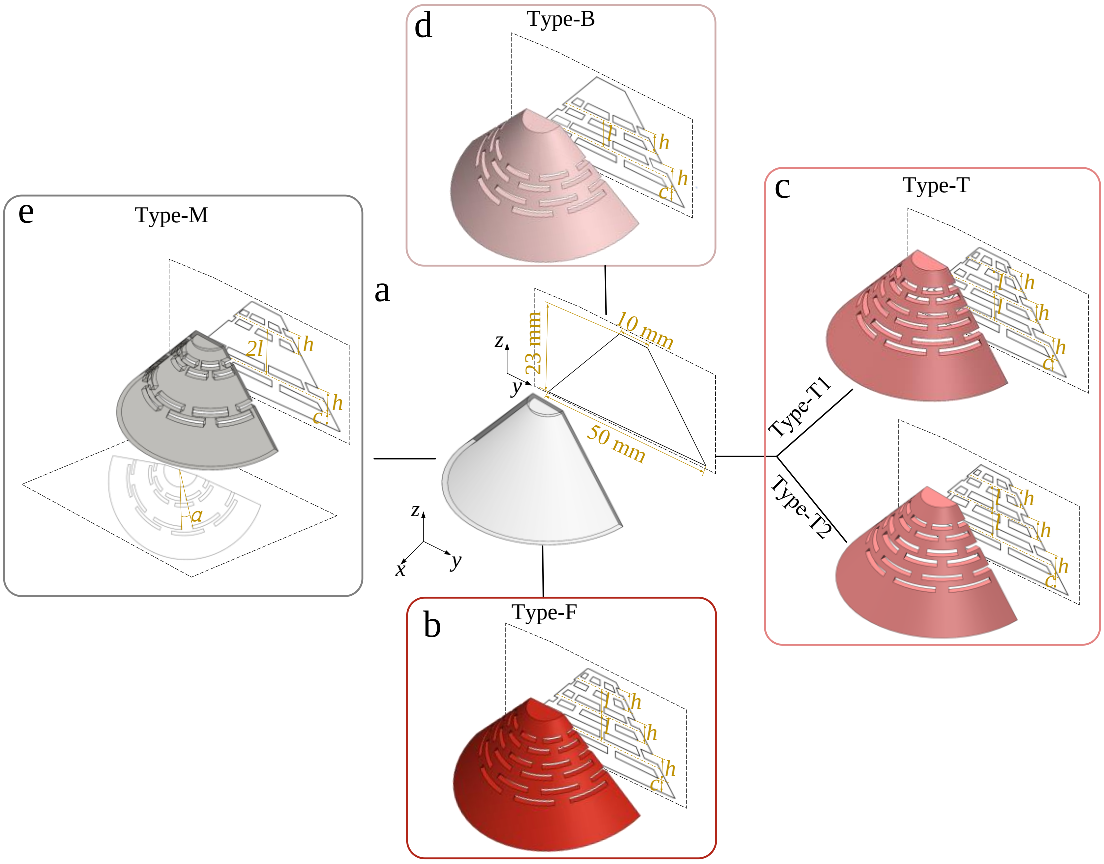

Figure S2. Motif design for perforated shell groups. A). The initial deep dome-pattern shell. b)-e). The motif design of Type-F, Type-T, Type-B and Type-M.

SI.3 Assembly of unit-cell

In the manuscript, metamaterials that can be mechanical force-driven and magnetic actuation supporting remote operation are shown. Due to differences in drive characteristics, the units are constructed and assembled differently, but the core components are all perforated shells. For the unit-cell supporting mechanical force-driven, the perforated shell is secured to the substrate by glue and is attached to the indenter and lid by bolt and nut (see Figure S3a). The height of the indenter is 8 mm and its presence allows the perforated shell to be compressed by the lid to the lowest steady state. For the unit-cell supporting magnetic actuation, two permanent magnets are arranged at the top and bottom of the perforated shell top and are tightly fixed to the perforated shell by magnetic force (see Figure S3b). The material of the permanent

magnet is N52 NdFeB. The magnetic attraction force of the two permanent magnets is much larger than the force of the magnetic field used to drive the perforated shell, In order to lower the center of gravity of the whole structure, the height of the permanent magnet on the lower side is 10 mm with a diameter of 8 mm and the height of the permanent magnet on the upper side is 4 mm with a diameter of 10 mm. The encapsulation shell is also attached to the substrate by glue for the protection of the unit and subsequent encryption of the information.

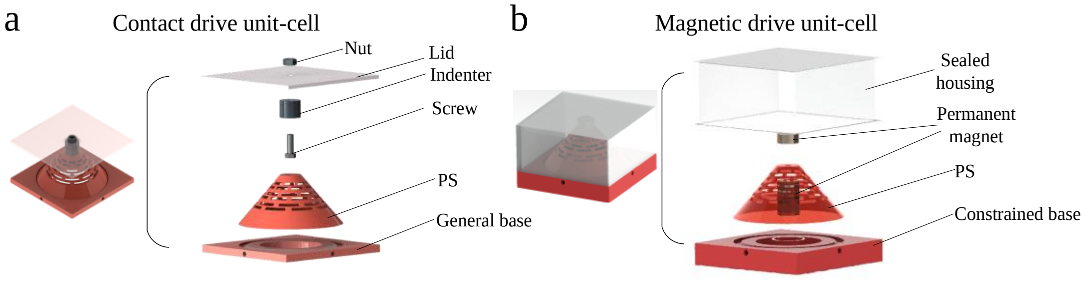

Figure S3. Components of an unit-cell a).Mechanical force-driven unit-cell. PS is connected to the base with glue. b).Magnetic-actuation unit-cell. PS and base, sealed housing and base are connected using glue.

SI.4 Force-displacement behavior of 3D printed PS

The force-displacement properties (especially the multistability behavior) of the four PSs are described and explained in detail here. Note that for all kinds of PS, the balance of the downward bending reaction force with the upward torsional reaction force leads to multiple stable configurations. When the Type-F PS is loaded, the hinge in the center of the third layer hole first enters the second stable state (State 1). Further increase of the downward displacement load leads to the curvature reversal of the first and second layers, thus reaching the third stable state (State 2). During unloading, the curvature of the second layer is again reversed and the curvature of the first layer remains unchanged, thus reaching the 4th steady state (State 3). For Type-T1 PS, during being loaded, the displacement could not be transferred to the third layer of beams due to the maximum

thickness of the beams in the second layer, which resulted in the first layer of the first layer jumping first and directly reaching State 3. As the applied displacement increases, the second and third layers almost simultaneously flip to reach State 2. The unloading process is consistent with the Type-F PS, which again goes through State 3. The multi-stable behavior of Type-T2 is more complex. The fact that it has the smallest thickness of the second layer results in the generation of a downward bending force that is not sufficient to counteract the upward torsional force generated when the third layer is flipped over, which results in the PS snap-back to its initial state (State 0) when the loading is removed. And the Type-T2 PS reaches its second stable state (State 2) only when the applied displacement is large enough to flip the second and third layers. The uninstallation process is consistent with the previous two PSs. Type-B PS has only the second and third layers of beams generated by perforation, and during the loading process, the third layer is first flipped to form the second configuration (State 1), while when the loading continues to increase, the lack of the first layer's support still prevents it from reaching State 2 of the other types of PS, despite the fact that the second layer is also flipped. For Type-M PS, it doesn't have a perforation in the middle therefore the middle part is very stiff, so only the snap-back behavior caused by the first layer flipping occurs during the loading process. It is worth noting that only Type-F and Type-T2 PSs have inconsistent load paths and unload paths; other types of PSs have the same load and unload paths.

SI.5 FE simulation of four kinds of PSs

Using SolidWorks 2018 and COMSOL Muliphysics 6.1, finite element models of individual BSUs were created to perform parametric design. Four kinds of PSs which possess different geometric parameter (i.e. Type-F, Type-T, Type-B and Type-M) made of the same material (TPU Nylon powder). According to the data obtained from uniaxial tension experiments, a linear elastic material model with Young's modulus of 135.45 Mpa and Poisson's ratio of 0.3 was built. The deformation of the PS under the apex

force is axisymmetric, so we only need to calculate a half of the PS. We focus on the t of the PS (within the range 0.6 mm $\leq t \leq$ 1.2 mm, with increments of 0.1 mm) and the polar angle (within the range 8° $\leq \alpha \leq$ 18°, with increments of 1°). To improve model convergence, dividing the grid ensure at least four elements through thickness and using the displacement control strategy to simulate the behavior of model buckle. Moreover, selected the "including geometric nonlinearities" option and a global damping value of $10e^{-4}$ was selected before calculating. The model mesh density of hinges is the largest because of its large deformation, while other regions are relatively less dense in order to improve computational efficiency. To suppress these so that a stable solution is obtained, the mid-point of the PS is constrained against sideways displacement through a symmetry condition. The global equation is determined by both the displacement at the indenter and the maximum displacement load and a linearly varying displacement parameter is introduced to simulate the increasing displacement. With the above method, the full steady-state position of the perforated shell during loading and unloading can be captured.

The simulation results for representative PSs visualize the differences between the various types of PSs (Figure S4). First, different types of PSs have different multistable properties, which have been elaborated in the main text. Second, different types of PS exhibit different stress distributions in each steady state, but the maximum stresses are relatively close. Third, the loading and unloading curves of the Type-T1 and Type-B perforated shells are consistent and the overall stress distribution is small. In addition, we find that the heights of the steady-state configurations of the various types of perforated shells are essentially the same, and their differences are negligible compared to the differences in the heights of the different state PSs. This lays the foundation for all types of perforated shells to be used for hierarchical storage of information. This is because thinner and fewer hinges make it easier for the PS to reach steady state.

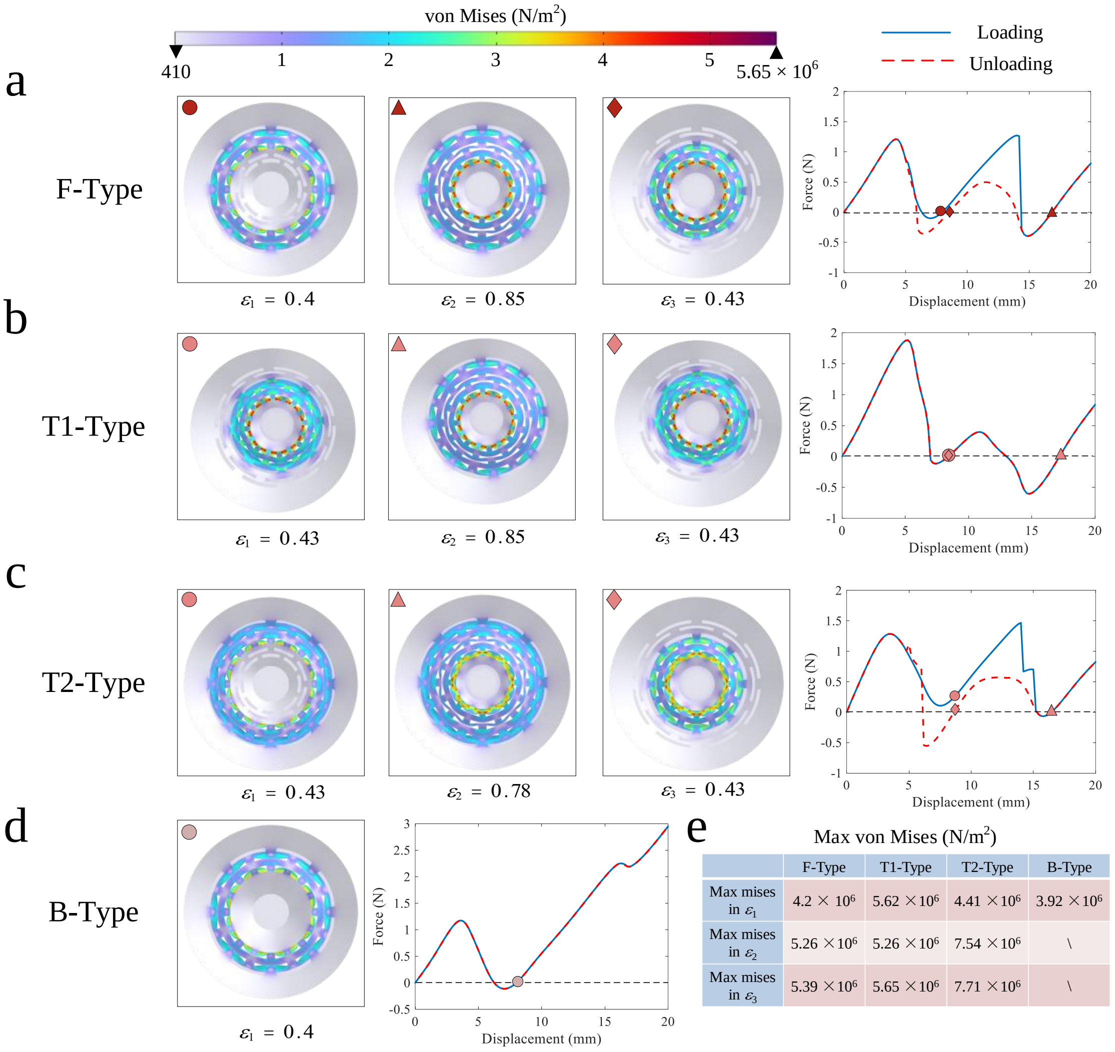

	F-Type	T1-Type	T2-Type	B-Type
Max mises in ε_1	4.2×10^6	5.62×10^6	4.41×10^6	3.92×10^6
Max mises in ε_2	5.26×10^6	5.26×10^6	7.54×10^6	\
Max mises in ε_3	5.39×10^6	5.65×10^6	7.71×10^6	\

Figure S4. Simulation and mechanical differences of four perforated shells. Snapshots of finite-element modelling of the stable state sequence about a).Type-F b).Type-T1 c).Type-T2 d).Type-B and loading and unloading curves. e).Maximum stresses for each type of PSs during loading.

Next, we performed uniaxial compress tests on various types of perforated shells, thus realizing controlled jumps in state through the selection of perforated shell categories (Figure S5). This can be combined with the design of the perforated shell parameters in the next section to control the displacement of its jump to the next steady state.

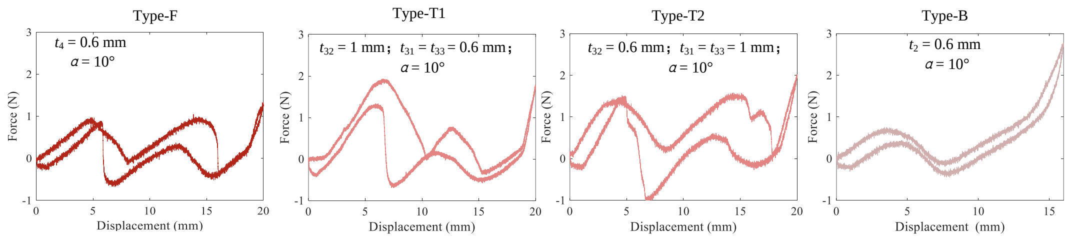

Figure S5. Differences in mechanical characterization tests of four types of PSs.

SI.6 Influence of geometrical parameters on various types of perforated shells

Parameterized simulations of PS groups are executed to provide guidance for building multi-tier information stores. Clearly, the geometrical parameters (i.e., t and α) not only affect the steady-state number of PSs, but also greatly influence their more detailed mechanical properties. The force-displacement simulation results of Type-B PSs with different geometric parameters (i.e. t and α) are shown in Figure S6. As t and α increase, the corresponding flexion force increases. On the other hand, when α increases, the PS arrives to the second steady state earlier under displacement-controlled loading, and this property is reversed with t. However, the height of the geometric configuration to reach the second steady state is essentially the same regardless of the variation of the geometric parameters. In addition, this combination of parameters determines the bistable properties of the Type-B PS,, and as t and α increase, the weaker the stability of the second steady state of the Type-B.

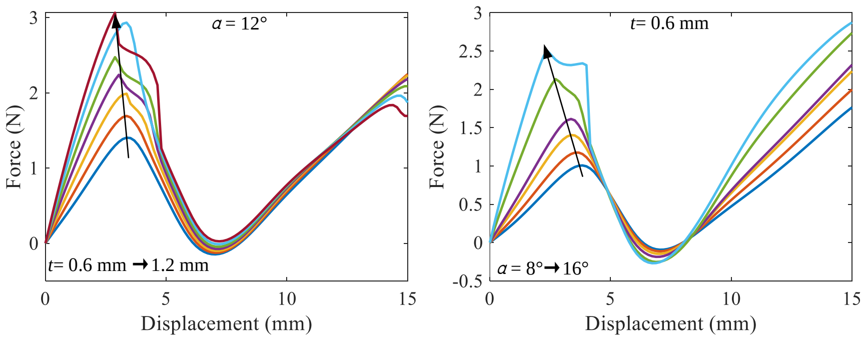

Figure S6. Influence of geometrical parameters on B-Type PS.

The effect of geometric parameters on the Type-T and Type-F PSs is similar to that of the aforementioned Type-B (Figure S7 and S8). However, for the more steady-state perforated shell group, we focus on the pattern of the two flexural forces as a function of geometric parameters. Specifically, the Type-T1 PS has essentially no larger first buckling force $F_{1\max}$ than second buckling force $F_{2\max}$, whereas the opposite is true for the Type-T2 PS, and the second buckling force $F_{2\max}$ is much larger than the first buckling force $F_{1\max}$ (Figure S7). And for Type-F PS, when t and a are set to smaller values, the first flexion force is smaller and vice versa (Figure S8a-b). The results show that the customization of the force-displacement curves can be achieved by selectively setting the geometrical parameters of the perforated shells, and, in addition, the design of perforated shells with similar flexural forces and different steady states makes it possible to construct perforated shell-based hierarchical information storage and decoding (Figure S8c-d).

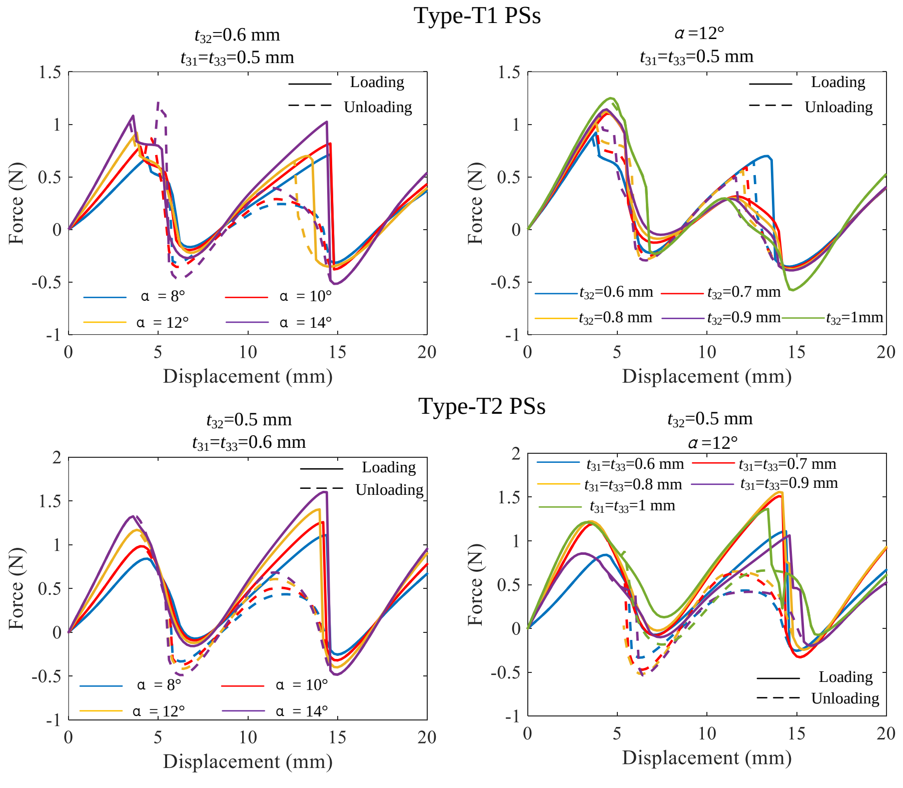

Figure S7. Influence of geometrical parameters on Type-T PS.

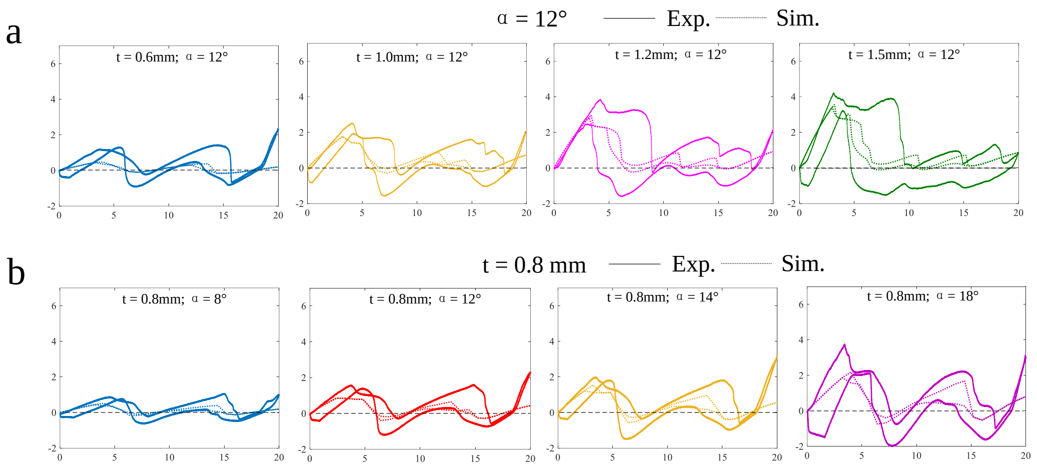

Figure S8. Influence of geometrical parameters on Type-F PS.

SI.7 Mechanical compression-driven metamaterial construction principles for encoding multilayer information

Properly designed perforated shells can experience multiple steady state phenomena when subjected to vertically oriented loads, and snap-through requires different displacement, which provides a viable basis for storing and reading information in metamaterials. Here, since most of the stored information is based on numbers and letters, we focus on a framework for building metamaterials with two states per layer of information. First, we show the reconfigurability of metamaterials. As shown in Figure S9, the metamaterial stores ’1 1’ in the first layer, ’H’ in the second layer, and reads it out through compression and release. The middle 4 units can be replaced by Type-B with other parameters by easily removing the connecting mechanism. Thereby, the information stored in the metamaterial changes to the first layer being ’H’ and the second being ’1 1’.

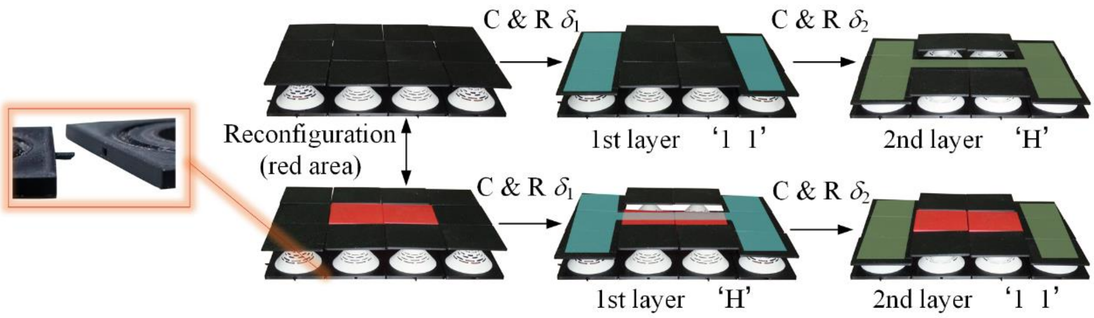

Figure S9. Demonstration of reconfigurable metamaterials.

Next, we elucidate the physical implementation of metamaterials to achieve multilayer information. A schematic of a metamaterial with multiple layers of information storage is shown in Figure S10a. The multilayered information can be encoded and stored in the metamaterial by specifying how each unit-cell changes state and further decoded by multiple steps of mechanical pressing. Each layer of information is separate and distinct, which requires that the unit must satisfy the state changes strategy shown in Figure S10b. In this strategy, the *n*th layer always has only two states, *n* and *n*-1. Each unit-cell crosses at most two steady states while keeping the state constant at least (e.g. 0→0→2→3) when it switches from layer *n*-1th to layer *n*th. In order to physically realize the tree diagram shown in Figure S10b, in the manuscript, we have presented seven samples for building metamaterials for two-layer (target information S→D) and three-layer information (target information S→D→U) storage. The force-displacement curves for each of the samples or their combinations demonstrate the physical realization of the state changes shown in the tree diagram. Here, we show snapshots of six samples used to store three layers of target information (Figure S10c) to visualize the physical implementation of the state switching process more intuitively.

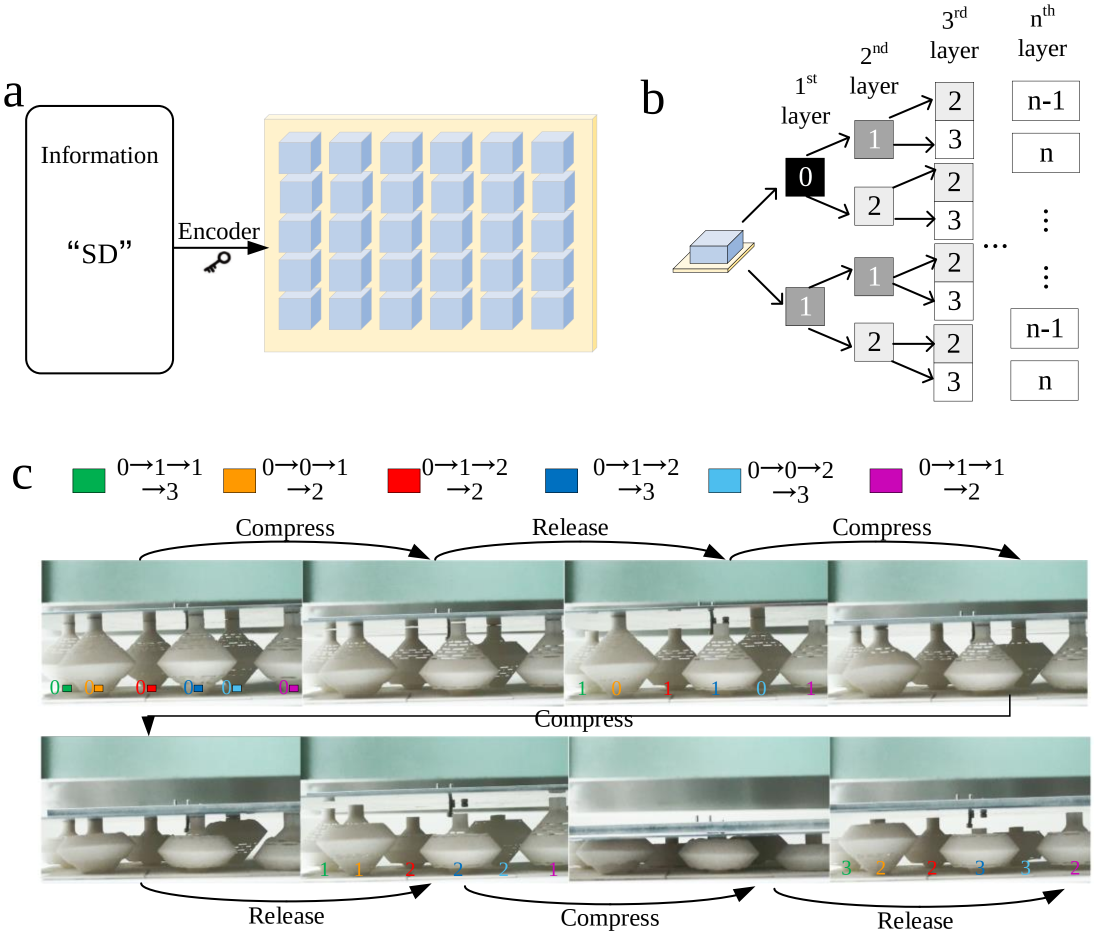

Figure S10. Metamaterials for information storage and reading. a).Schematic diagram for storing information in multiple layers. b).Principle diagram of multi-layer information design. c).Snapshots of deformation sequence of the six samples.

Based on the main text, we also demonstrate the application of metamaterials to generate multidimensional information through one-step loading. A single perforated shell can generate up to three different states during loading, and 2 perforated shells in series can generate up to 5 different states. As a result, metamaterials can enable the transformation of multiple multidimensional shapes such as domes, saddles, cylinder, (Figure S11) and so on.

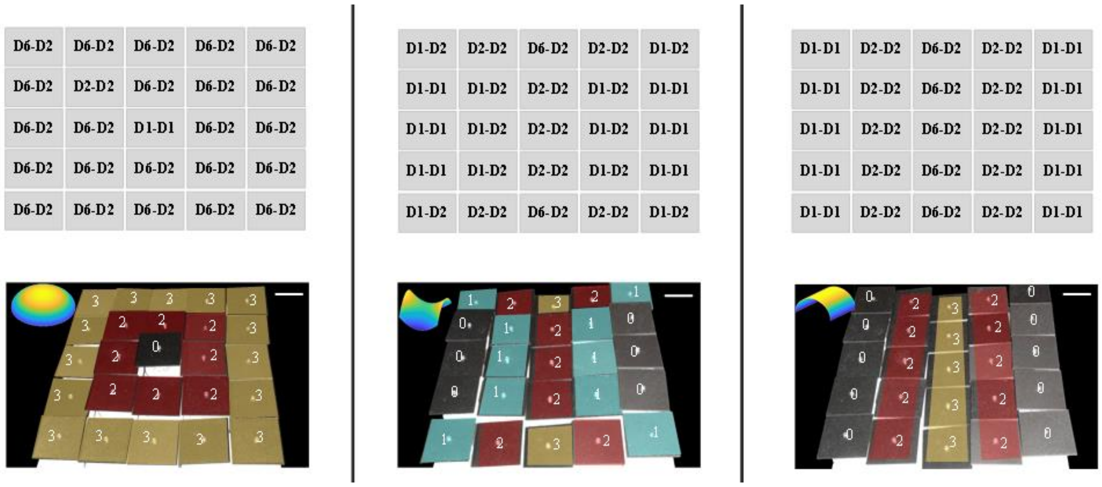

Figure S11. Multidimensional information for single layer graphic (scale bar = 3 cm).

SI.8 Cyclic repeatability testing of representative PSs

To analyze the reproducibility and stability of metamaterials after multiple "compression-releases" operations, we performed six continuous force-displacement tests on each of the 7 samples (Sample D1-D7) shown in the Figure 3a. All samples are loaded and unloaded at a speed of 1mm/s. In fact, the stability of PS in each state is closely related to its force-displacement curve. Their force-displacement curves of all the samples show good agreement, which means that the stability of the samples in different after states is almost unaffected after a series of cycles (Figure S12a). Next, based on the above test results, we verify that metamaterials can reproducibly achieve multilayer information storage. We introduce $\delta_{i\min}$ to describe the minimum displacement required for the PS to switch to next stable state (State i). Figure S12b counts the displacement $\delta_{\min}$ of switch to the next state every time for the six samples. From the results of several tests, we obtained (i). $\delta_{1\min}^{D3} > \delta_{1\min}^{D7} = \delta_{3\min}^{4} > \delta_{1\min}^{D2}$ and $\delta_{1\min}^{D6} > \delta_{1\min}^{D7}$; (ii).Sample D5 is always absent State 1; (iii). $\delta_{2\min}^{D5} > \delta_{2\min}^{D7} = \delta_{2\min}^{D4} = \delta_{2\min}^{D6}$. Meanwhile, the coefficient of variation (CV) was introduced to describe the degree of dispersion of $\delta_{i\min}$. The CV for each sample can be obtained by dividing the standard deviation by the mean value. From Figure S12b, it can be obtained that $\delta_{3\min}$ of Sample

4 has the largest *CV* (*CV* =2.92%). These results imply that our encoder and its combination design strategy is still effective after six consecutive compression-release cycles. The PSs still demonstrate the initial mechanical behaviors which is used to design the encoder, experimentally demonstrating its repeatability and robustness.

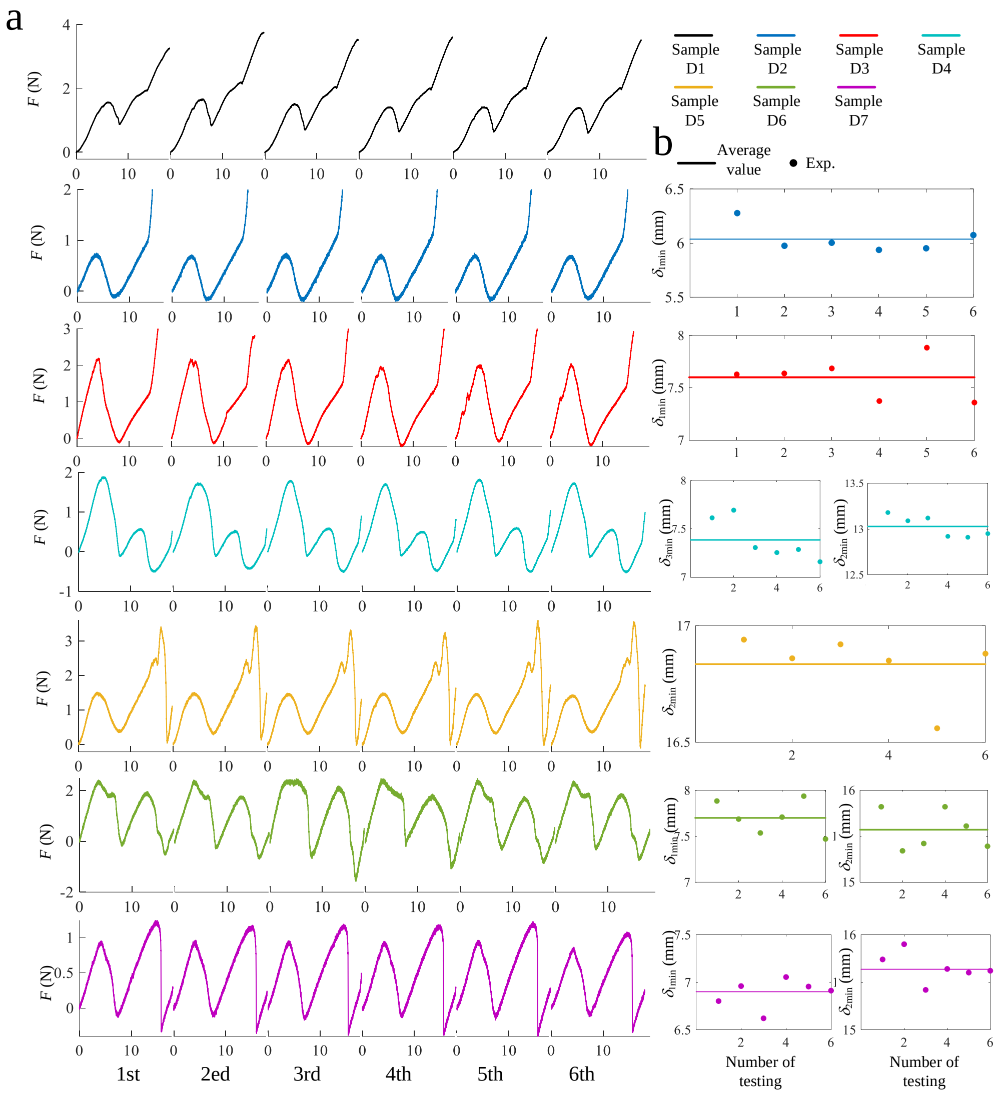

Figure S12. Force-displacement behaviors of 7 basic samples during repeated loading tests. a) Each sample is tested six times to obtain the corresponding force-displacement curves. b) Repeatability test results for the minimum displacement $\delta_{i\text{min}}$.

SI.9 Impact of loading speed on four PSs

We also investigated the effect of loading speed on the force-displacement characteristics of perforated shells during uniaxial compression tests. For this reason,

we used different loading speeds (i.e. 0.5 mm/s, 1 mm/s, 1.5 mm/s, and 2 mm/s) to quantify this effect. The experimental results show the effect of loading rate on the mechanical properties of the four PS is not significant (Figure S13). Additionally, the number of stable-state of PSs n does not vary with loading speeds. The above results show that encoding and decoding of metamaterials is independent of the loading speeds. Therefore, the state transition time of the metamaterial is proportional to the loading speeds. For illustrative purposes, all units are loaded uniformly and the mechanical loading speed was 1 mm /s in this paper.

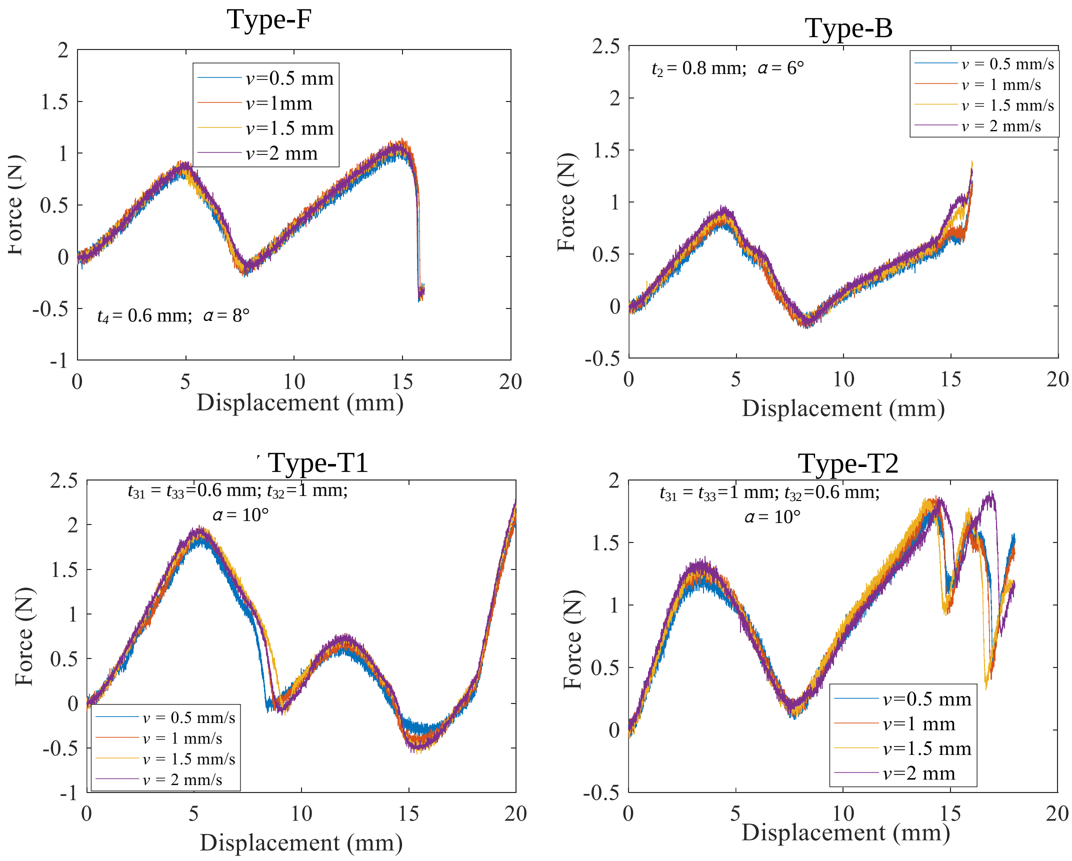

Figure S13. Effect of loading velocity on the mechanical behavior of various types of perforated shells.

SI.10 Magnetic field generated by the electromagnetic coils

In this study, the magnetic field is generated by two coaxial coils to which opposite currents are applied. In order to be able to effectively program metamaterials, we follow

two main principles when designing the magnetic field drive. The first being that the force acting on the permanent magnet needs to be large enough to trigger the jump of the perforated shell to reach another steady state. The second is that there must be a reasonable physical quantity that does not affect the magnetic field properties to be used to program the metamaterial. The third is to make the force of the magnetic field region on the permanent magnet as constant as possible (i.e., the spatial region where the magnetic force is constant is as large as possible), which is the key to controlling the realization of state switching in each unit-cell. In order to solve the above problems, we have theoretically derived and simulated the magnetic field generated by an electromagnetic coil under constant current application.

According to the Ampere model, the gradient of the manetic field near the midpoint of the axes of the two coils configured in this way is constant, and therefore the force on the permanent magnet is also constant. This is a critical step for us to encode and read metamaterials with different currents. According to the Biot-Savoie law, the magnetic field along the central axis produced by two coaxial electromagnetic coils can be obtained as

$$B(z)=\frac{A}{\sqrt{((z-1+\frac{h}{2})^2+R^2)^3}}-\frac{A}{\sqrt{((z-1-\frac{h}{2})^2+R^2)^3}} \tag{1}$$

Where h is the perpendicular distance between the two coils, R is the average radius of the coil and $A=\mu_0 NIR^2$ (μ_0 is the vacuum permeability, N is the number of turns of the coil and I is the current applied to the coil).

Further, it is assumed that the intrinsic magnetic moment of the permanent magnet is $\mathbf{m}$. Then the energy of the permanent magnet in the magnetic field is

$$W=-\mathbf{m}\bullet\mathbf{B} \tag{2}$$

For the present study, we only consider the magnetic field strength in the z-direction on the axis, and the above equation can be simplified as

$$W=m_z\left(\frac{A}{\sqrt{((z-1+\frac{h}{2})^2+R^2)^3}}-\frac{A}{\sqrt{((z-1-\frac{h}{2})^2+R^2)^3}}\right) \tag{3}$$

Where h_m (M is the magnetization strength, h_m、 r are the height and radius of the permanent magnet, respectively.) and the magnetic force on a permanent magnet can be obtained by

$$F = -\frac{\partial W}{\partial z} = m\frac{\partial}{\partial z}B(z) = \frac{3mA}{2}\left[\frac{3(2z+h)}{((z+\frac{h}{2})^2+R^2)^{\frac{5}{2}}} - \frac{3(2z-h)}{((z-\frac{h}{2})^2+R^2)^{\frac{5}{2}}}\right] \quad (4)$$

Next, as mentioned previously, the parametric design of the electromagnetic coils was executed. We wanted the area of constant magnetic force to be as large as possible, which is more conducive to controlling perforated shell actuation or reset. After simulation and analysis, the size of the constant force region is independent of the current applied to the coil and the number of turns in the coil. Therefore, this further demonstrates that by regulating the current to program the metamaterials. Further, when the two coils are vertically separated by an average radius satisfying the $h = \sqrt{3}R$, the region of constant magnetic force in this field is always the largest. Considering the height of our metamaterial itself, we ended up setting the vertical distance h and radius of the coil R to 80 mm and 46.2 mm (Figure S14). Figure S15 shows a snapshot of the PS switching to another state under magnetic drive. The results show that the response time of the PS is less than 80 ms.

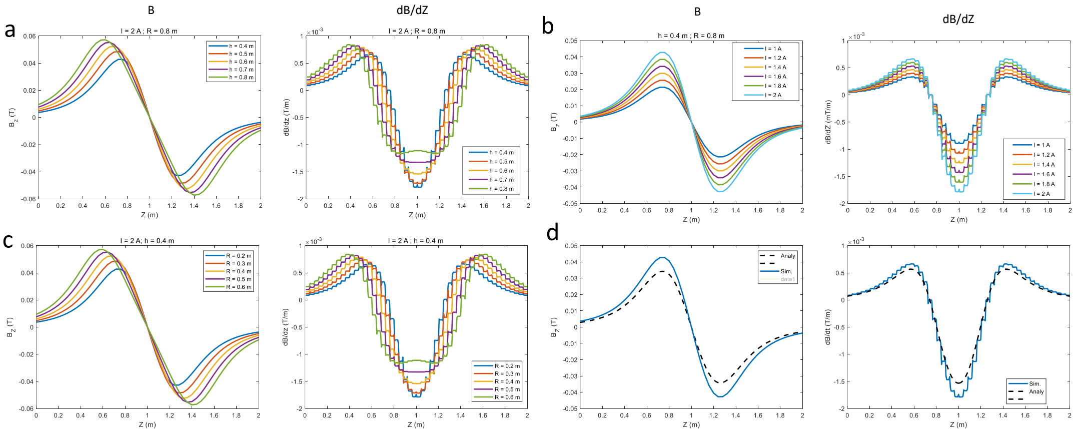

Figure S14. Simulation of magnetic fields generated by Helmholtz coils. Relationship between the generated magnetic field and the distance a) current b) and radius of the

coil. c)-d).Comparison of simulation results and theoretical solutions.

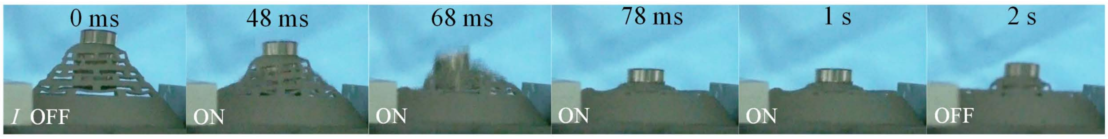

Figure S15. The snapshot of the PS switching to another state under magnetic drive.

SI.11 Parametric design of perforated shells for magnetic field actuation

In the manuscript, we point out that the magnetic field remote reading of metamaterials is based on the force control strategy of permanent magnets. The main advantage of magnetic field actuation over mechanical compression actuation is the ability to remotely switch and read information even when the metamaterial is encapsulated. On the other hand, since the force controls the perforated shell switching rather than the displacement control, the situation where the perforated shell switches directly from State 0 to State 2 no longer requires the involvement of Type-T PSs. The magnetic force only needs to reach the maximum buckling force of the perforated shell to realize it (regardless of which one of F_{1max} and F_{2max} is larger). Unfortunately, this leads to a new dilemma: when the buckling force relation of the perforated shell is $F_{1max} \geqslant F_{2max}$, the magnetic drive cannot traverse all steady states of the perforated shell, especially the second steady state is crossed. According to the detailed analysis, there are fewer perforated shells in the Type-F PSs that meet the requirements (Figure S16), and there are cases of crossing the intermediate steady state in the Type-T2, so more PS types are needed to extend the programmable range of magnetic field drives.

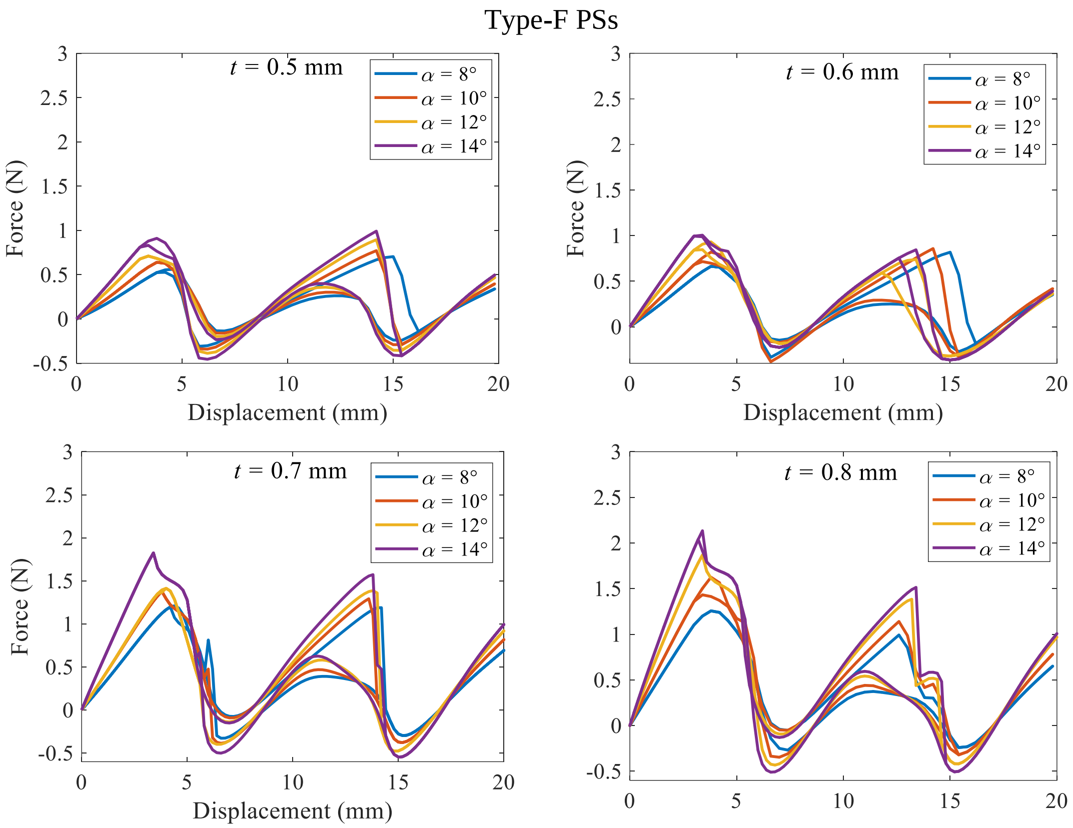

Figure S16. Force-Displacement Behavior in Type-F PS (t is from 0.6 mm to 1 mm and α is from 8° to 18°).

Here we propose irregular PS configurations (Type-Fi) based on Type-F PSs. When α is taken to be 10° , the Fi-type has a more pronounced gap between the two flexion forces than the Type-F (Figure S17). This is due to the change in the dominant deformation of the PS to reach the steady state when the Type-F of the same parameter is changed to Type-Fi. For Type-Fi, which has a ratio of two buckling forces between Type-F and Type-T2. In essence, this combination of methods enables different hopping sequences of 0→1→2, 0→0→2, and 0→1→1 in a constant magnetic field. At the same time it does not affect the hopping of the perforated shells of 0→2→2, thus this greatly enriches the programmable space of magnetically driven metamaterials.

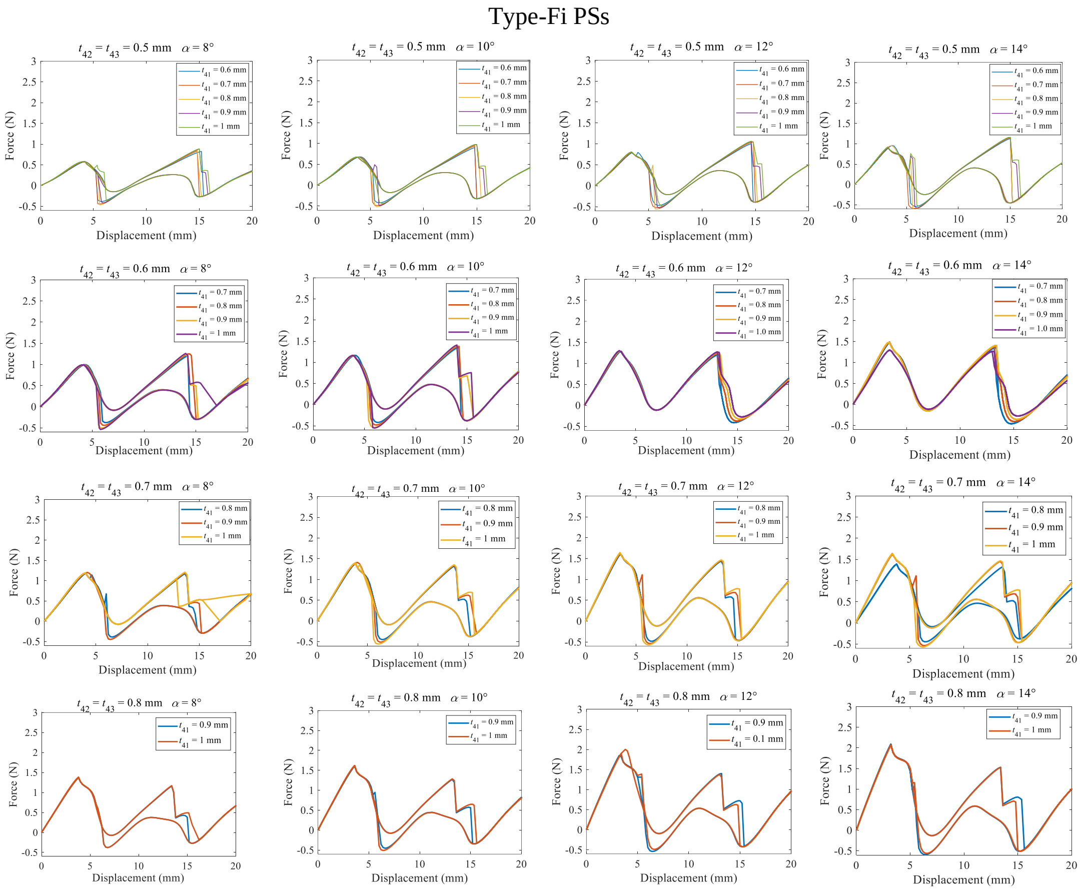

Figure S17. Force-Displacement Behavior in Type-Fi PS (t is from 0.6 mm to 1 mm and α is from 8° to 18°).

SI.12 Mechanical stability of different types of perforated shells

Finite element simulations are performed to investigate the effect of the layer number and non-uniform parameter settings on the stability of the structure. For the potential energy-displacement curve of the PS, the valley (State i) corresponds to the energy E_{vi}, while the peak corresponds to the energy E_{pi} (to switch from State i to another state, Figure S18a(i)). Here, we quantify the mechanical stability of the structure in terms of ΔE_i ($\Delta E_i = E_{pi}$-E_{vi}). A higher ΔE_i means a more stable configuration. Compared to the Type-B PS with the same geometric parameters, the Type-F PS has a lower ΔE_i, which implies that the increase in the number of layers weakens the stability of the structure in State 1 (Figure S18a(ii)). However, the effect

of non-uniform parameters on the mechanical stability of PS is complex because they have an effect on the stability of all three non-initial configurations. For State 1, The reduction of t_{32} in the perforation of the middle layer similarly weakens the stability of the PS in State 1 (Figure S18a(iii)), consistent with the stability exhibited by the T2-Type PS in the manuscript. For State 2, a decrease in the projection height for top and bottom perforations (t_{31} & t_{33}) then increases the stability of State 2 (Figure S18b(ii)), while the reduction of t_{32} in the middle layer still weakens the stability of State 2 (Figure S18b(iii)). In State 3, Type T1 and T2 PSs are more stable than Type-F PS with parametrically homogeneous when the projection height t_{32} is lower. While as t_{32} increases, the F-type PS is more stable in state 3 (Figure S18c(ii)—(iii)). Finally, since Type-M is a monostable structure, its stability in the initial state (State 0) is clearly higher than the other three PSs.

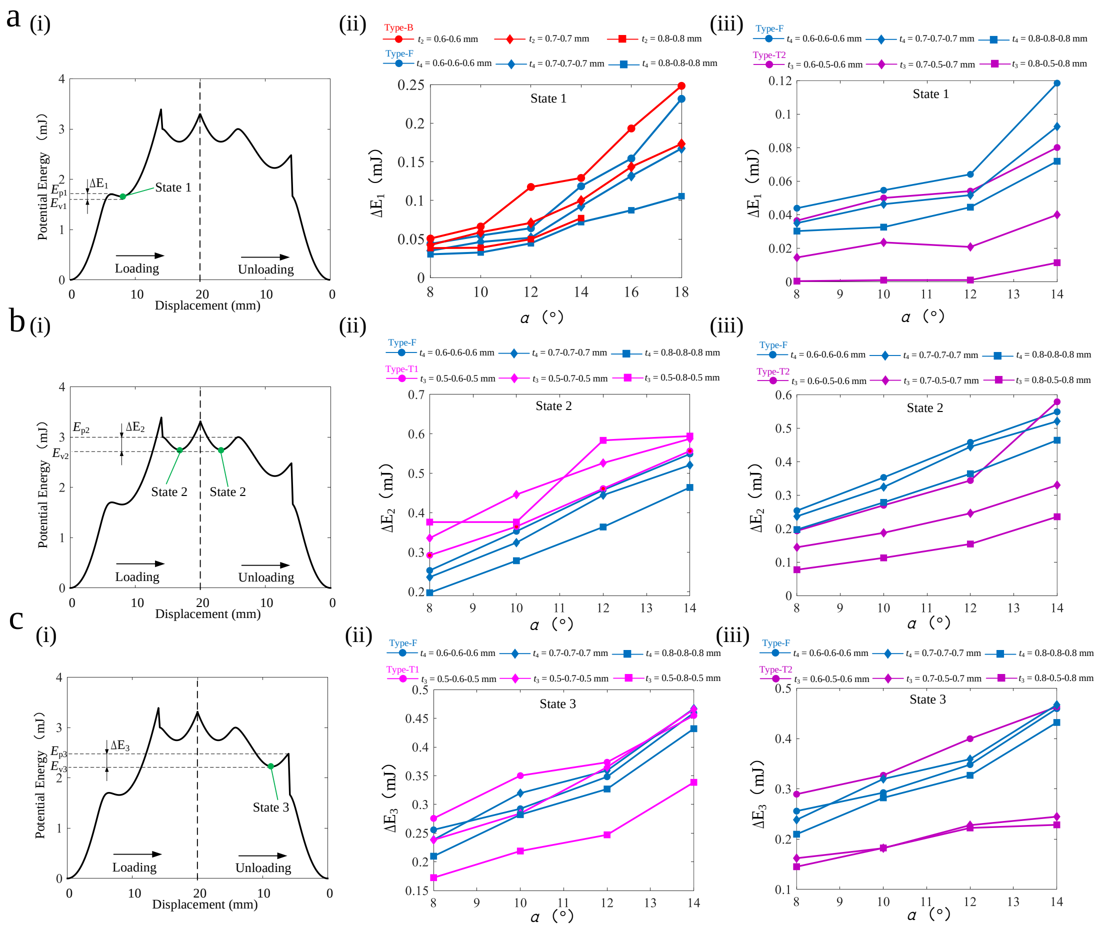

Figure S18. The effect of the layer number and non-uniform parameter settings on the

stability of the PS. a).State 1. b).State 2. c). State 3.

SI.13 Constrained base design for electromagnetic actuation decoupling

Resolving the magnetic coupling of adjacent units is the key to achieving a remote drive. We observe that PS exhibits additional stable state in both State 1 and State 3. PSs exhibit bending deformation under non-axial pressure and reaches extra stable configurations in this configuration (State 1-1 and State 3-1), as shown in Figure S19. While they are subjected to axial pressure, will transform to State 2. Here, we refer to State 1 and State 3 as bifurcated states. However, this phenomenon is not observed in States 0 and State 2. Therefore, the problem turns to how to limit the non-axial deformation of the PS in States 1 and 3. We design the constraint base to constrain the non-axial deformation of the PS, however, in order for the PS to traverse all stable states, the PS in State 0 will not be constrained. This is because too high a restriction hole in the base can cause the PS to lose State 2.

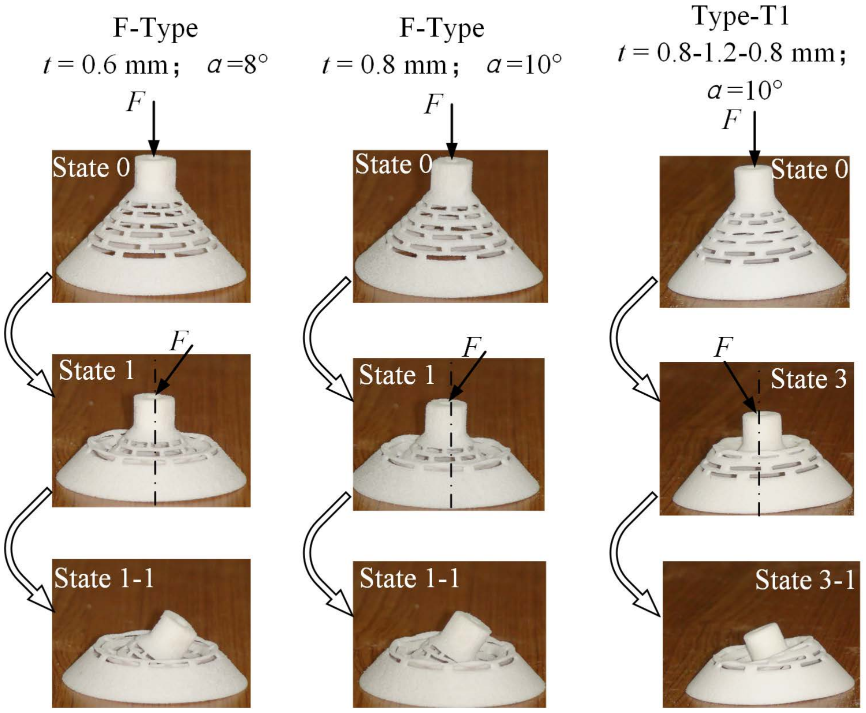

Figure S19 Additional stable state of PSs.

The design parameter of the constraint base of the magnetic drive unit is the distance b between the top of the constraint hole and the fixed plane of the PS. When b is too small, the constraint base cannot act as a constraint as it should, and when b is too large, the PS cannot traverse the full range of stable states (especially, State 2). Here, two constrained bases (b=1mm and b=3mm) are used to test the effect of magnetic decoupling. As shown in Figure S20, the PS marked in red in the center is on the central axis of the solenoid coil and is regarded as the target unit-cell for magnetic programming. Both forward and reverse current inputs will only result in state switching of the target unit without affecting the other units as b=3mm. And when b=1mm, the electromagnetic force still affects other units around the programmed target.

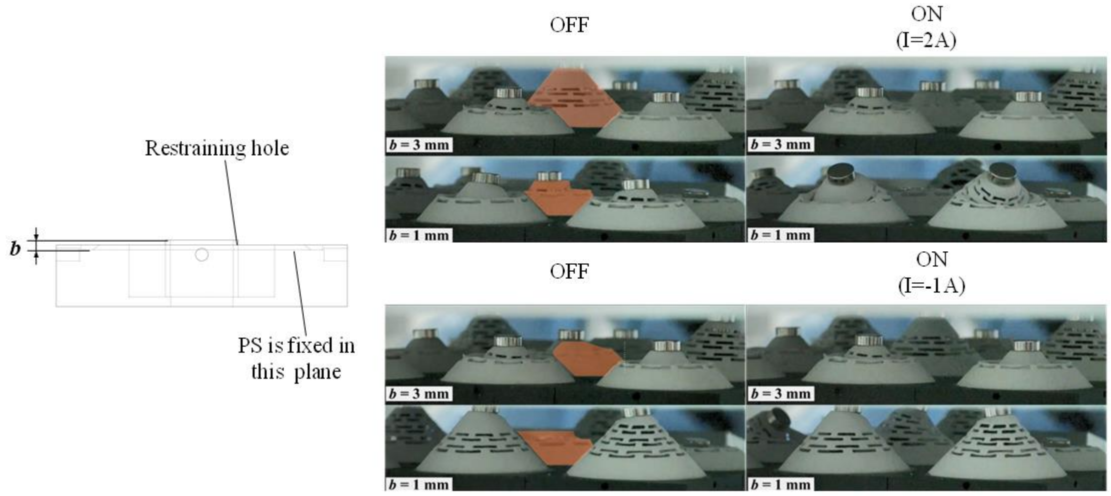

Figure S20. Parametric design and demonstration of constrained bases

SI.14 Principle of realizing information encryption in metamaterials

Next, we will further utilize metamaterials as a information encryption material (IEM) to purposefully encode different information onto each unit-cell without introducing any additional electronic components. Figure S21a shows the working mechanism for the designed IEM. By rationally designing each PS unit, the metamaterial is decrypted by applying a decoder (i.e. suitable current to the external coil). Under the excitation of other amplitude currents, the metamaterial will display

unrecognizable information. Given the above requirements, we introduce the concepts of both functional and confusion units in IEM. The former is used to encode valid information, while the latter will undergo unanticipated deformations when motivated by non-decoded inputs (Figure S21b). Numerically, we can explore the influence of the geometry of the PSs on both (I) confidentially and (II) unique solution of the IEM. For (I), to ensure confidentially, some units of the IEM must have more states in response to all stimuli. And for (II), IEMs appear valid only in the presence of a decoder, while information produced under other excitations are not recognizable (e.g., letters and numbers). In addition, we have designed two 4×4 arrays to realize single-layer and double-layer information encryption according to the above principles (see Figure 5a-d in manuscript).

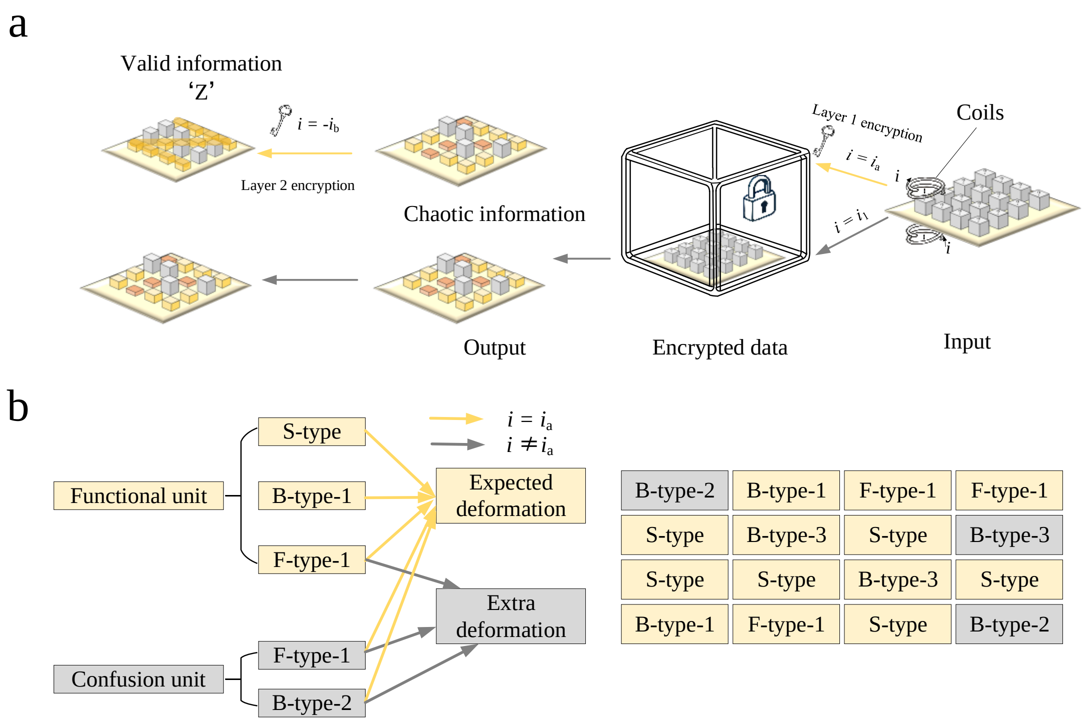

Figure S21. PS metamaterials are used for two-layer encryption. a). Schematic diagram of metamaterials to realize information encryption. b). In accordance with the encryption principle, functional units (brown block) and confusion units (grey block) are introduced into the metamaterial at the same time.

SI.15 Multi-step mechanical logic gate construction principle

To construct a two-step mechanical logic gate, it is essential to ensure that the perforated shells exhibit three steady states during the driving process. Analyzing various types of PSs reveals that the number of PS steady states varies significantly during the loading process, while it remains constant during the unloading process, consistently undergoing three steady states. Therefore, we select State 2 as the initial state of the mechanical logic gate.

The mechanical signal from input to output is transmitted through the energy released by PS state switching via a connecting beam angled at 45° to the horizontal plane. When the energy released by a single PS at the input is insufficient to supply the energy required for the PS state switch at the output, but the combined energy of two PSs is adequate, the system functions as an AND gate. Conversely, when the energy released by one PS at the input is sufficient to supply the energy needed to switch the state of the PS at the output, and the connecting beams are constrained to make the PS at the output switch to a steady state, the system functions as an OR gate.

To guide the design of two-step mechanical logic gates, we conducted finite element analysis to study the effect of geometric parameters on the energy barriers generated by various types of PSs (Figure S22a). Using the force-displacement characteristics of these PSs, subsequent simulations were performed to predict their properties during unloading. For the Type-F PS, the energy released during the second state transition increases monotonically with t and α (Figure S22b). The energy released during the second transition of the Type-Fi follows a similar pattern. However, the effect of α on the energy released during the first transition is more complex. When α is small, the released energy is not very sensitive to changes in α, but when α is large, the released energy becomes much more sensitive to changes. The energy released by the PS during the first transition reaches its maximum value when t_{42} and t_{43} are approximately 0.62 mm (Figure S22c-d).

For the Type-T PS, the behavior is even more complex. It is evident that the energy

characteristics of the Type-T1 PS are linearly related to t. Specifically, when t_{32} decreases and t_{31} and t_{33} increase, the energy released by the PS during the first transition also increases (Figure S23a). Conversely, the energy released during the second transition increases with t_{32} (Figure S23b). These properties enable the construction of logic gate cells with adjustable functions. For the Type-T2 PS, when t_{31} and t_{32} are equal to 1 mm, the perforated shell exhibits only three steady states, resulting in a sudden increase in released energy near the 1 mm region (Figure S23c-d). This characteristic further divides the energy properties of the Type-T2 PS into two distinct regions.

Having demonstrated that the energy barrier for a transition wave to propagate can be controlled by tuning t and α, we now illustrate how functional devices can be designed by strategically arranging the PSs. It is critical to note that the pulse propagates independently of its initial conditions, allowing it to be manipulated through entirely local geometric changes. This phenomenon can be attributed to the excessive damping of the system, wherein only a specific signal that matches the local geometric parameters can propagate to the next node. Due to the inherently unidirectional transition from the high-energy state to the low-energy state that each individual bistable unit undergoes during propagation, an external energy source is required to reset the bistable elements to their higher-energy state for additional propagation events.

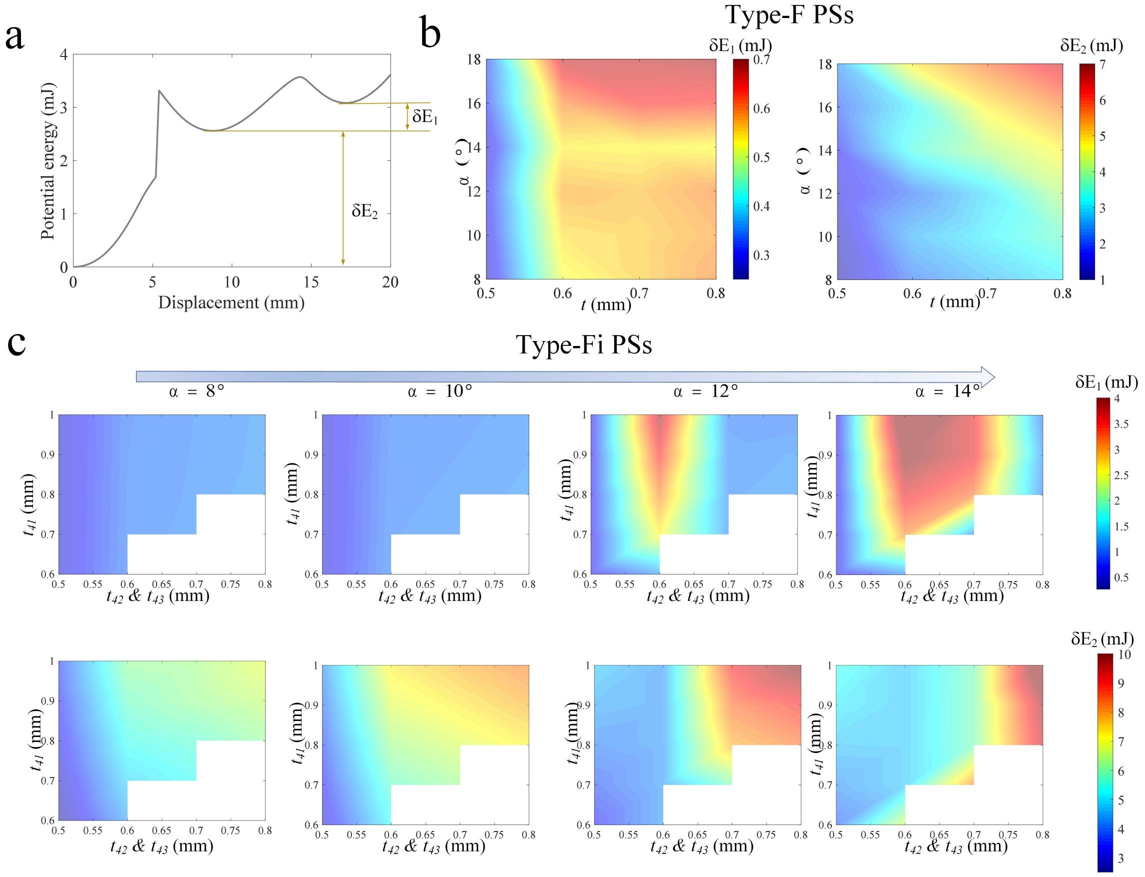

Figure S22. Energy released during unloading as a function of geometric parameters for Type-F and Type-Fi. a) Energy profile of the perforated shell when switching from state 2 back to the initial state. b) Energy released when Type-F PS returns to its initial state as a function of geometric parameters. c) Energy released when Type-Fi PS returns to its initial state as a function of t_{41} and t_{42} when the α takes on different values.

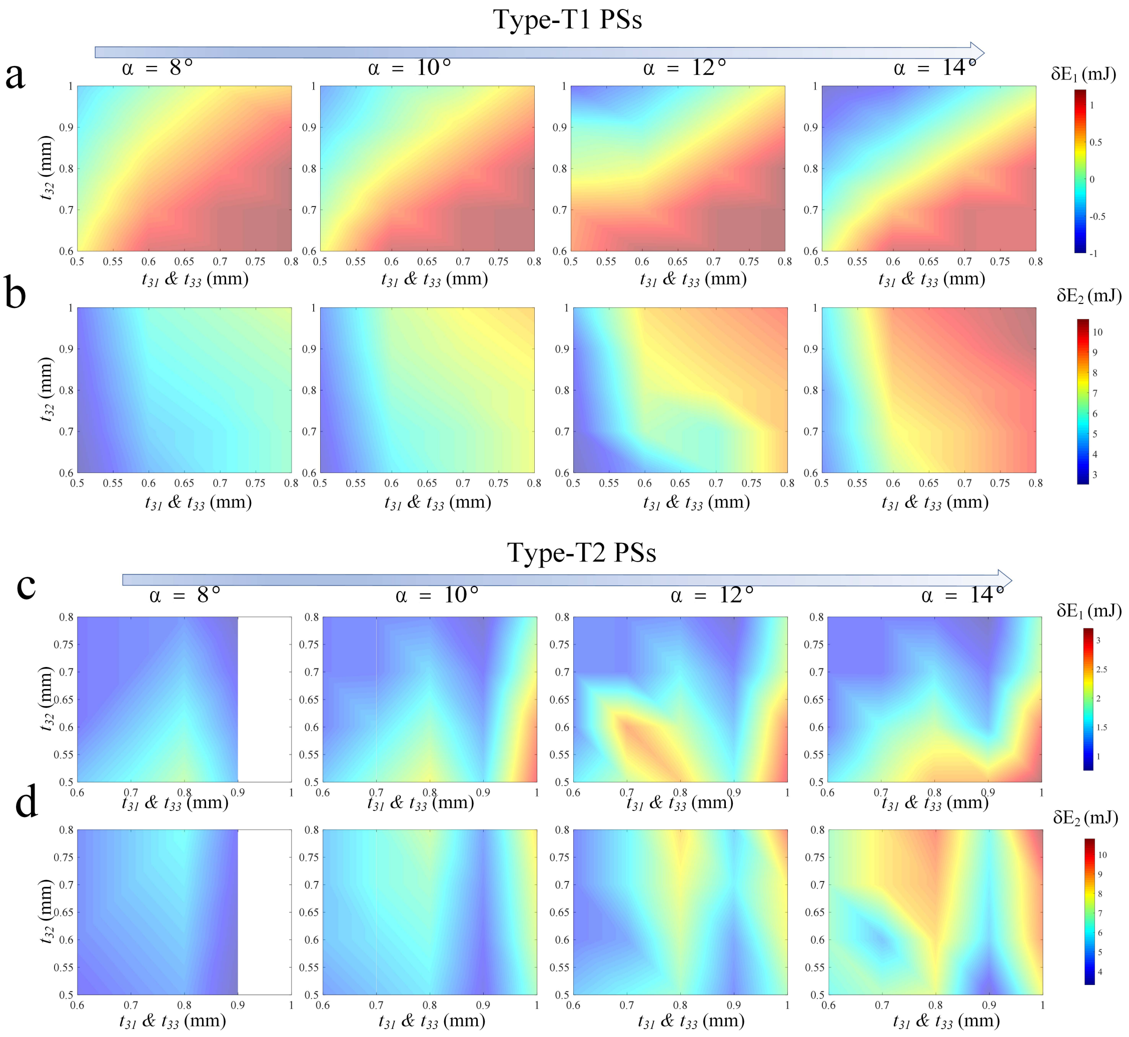

Figure S23. Energy released during unloading as a function of geometric parameters for Type-T1 and Type-T2. a)-b). Energy released when Type-T1 PS returns to its initial state as a function of t_{31} and t_{32} when the α takes on different values. c)-d). Energy released when Type-T2 PS returns to its initial state as a function of t_{31} and t_{32} when the α takes on different values.

SI.16 Influence of geometrical parameters of series-connected PS units on compressive stiffness

In the manuscript, we demonstrate the functionality of PS cell-based metamaterials

in terms of reprogrammable dynamic information transfer capabilities. Essentially, this is achieved by switching the PS state resulting in a significant change in stiffness. Here, we use a unidirectional four-stable metamaterial formed by connecting Type-F PS and Type-B in series to realize the switching of force transfer modes in the low frequency range (e.g., frequencies below 30 Hz). In the main text, we have shown demonstrations of metamaterials being used to change height without changing the force transfer properties as well as changing the force transfer properties by changing height. Further, we quantitatively demonstrate how a customized force-transfer metamaterial can be reverse-engineered. For this purpose, the relationship between stiffness and the geometrical parameters was analyzed for each type of PS. Since the force transfer properties are closely related to the load, all stiffness measurements in this study were obtained under a 200 g load. (Figure S24a-b). The stiffnesses k_1 and k_2 of B-Type PS increase with t and α (Figure S24c). The trend of PS stiffness for other types is similar (Figure S24d-f). We note that for Type-T2 and Type-F PSs, there is little difference between their stiffnesses k_2 and k_3. Thus, in terms of changing the mode of force transfer (Figure S24e-f), this structure has three modes instead of four. On the other hand, the Type-B PS exhibits the largest difference between the two stiffnesses, while the Type-T2 PS has the smallest difference. The remaining two types fall in between these extremes. This suggests that the combinations of PSs in metamaterials can be made more or less adjustable by varying their geometrical parameters, enabling the creation of customized force transfer metamaterials.

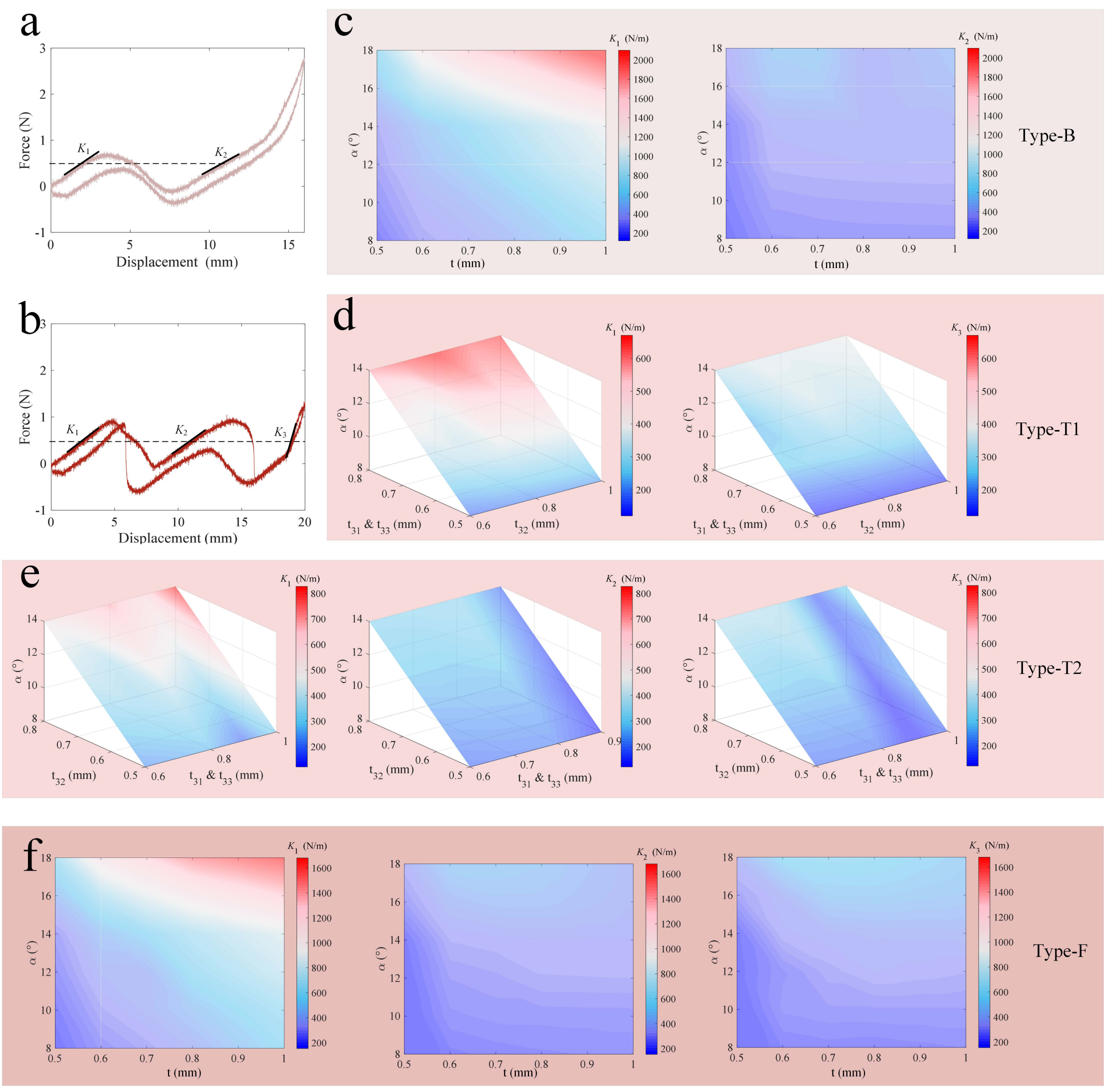

Figure S24 Effect of geometrical parameters on the compressive stiffness of various types of perforated shells. a)-b). Schematic diagram of various stiffness parameters. c).Type-B. d).Type-T1. e).Type-T2. f).Type-F.

SI.17 Dynamic information transfer in customized frequency bands

We also demonstrate the inverse design of metamaterials for signal amplification in specific frequency ranges. Figure S25a shows the transmissibility at frequencies from 1 to 30 Hz for Mode 1 and Mode 2 under the weight of 200 g. The results show that the resonance peak of Mode 1 (9 Hz) is significantly smaller than that of Mode 2 (11 Hz) and Mode 3(13 Hz) due to the difference in compression stiffness. The isolators at Mode

1 can isolate vibrations when the frequency is higher than 13 Hz. As for Mode 2, vibration isolation requires that the frequency is higher than 19 Hz. Moreover, considering that the stiffness of Mode 2 decreases significantly as the strain increases, the transmissibility of Mode 2 under different loads is shown in Figure S25b-c. It can be seen that the resonance peak under the mass of 390 g is smaller than that under 90 g. Mode 1 isolators can isolate vibration at frequencies above 13 Hz. For modes 2 and 3, isolation is required above 19 Hz. In addition, considering that the stiffness of Mode 2 decreases significantly with increasing strain, the transferability of Mode 2 under different loads is shown in Figure S25d-e. Combined with Fig. 6 in the main text, it can be seen that we can customize the force-transmitting metamaterial to have the greatest transfer efficiency for a given frequency. Based on the above analysis, we show more examples of multi-step adaptive drives (Figure S26).

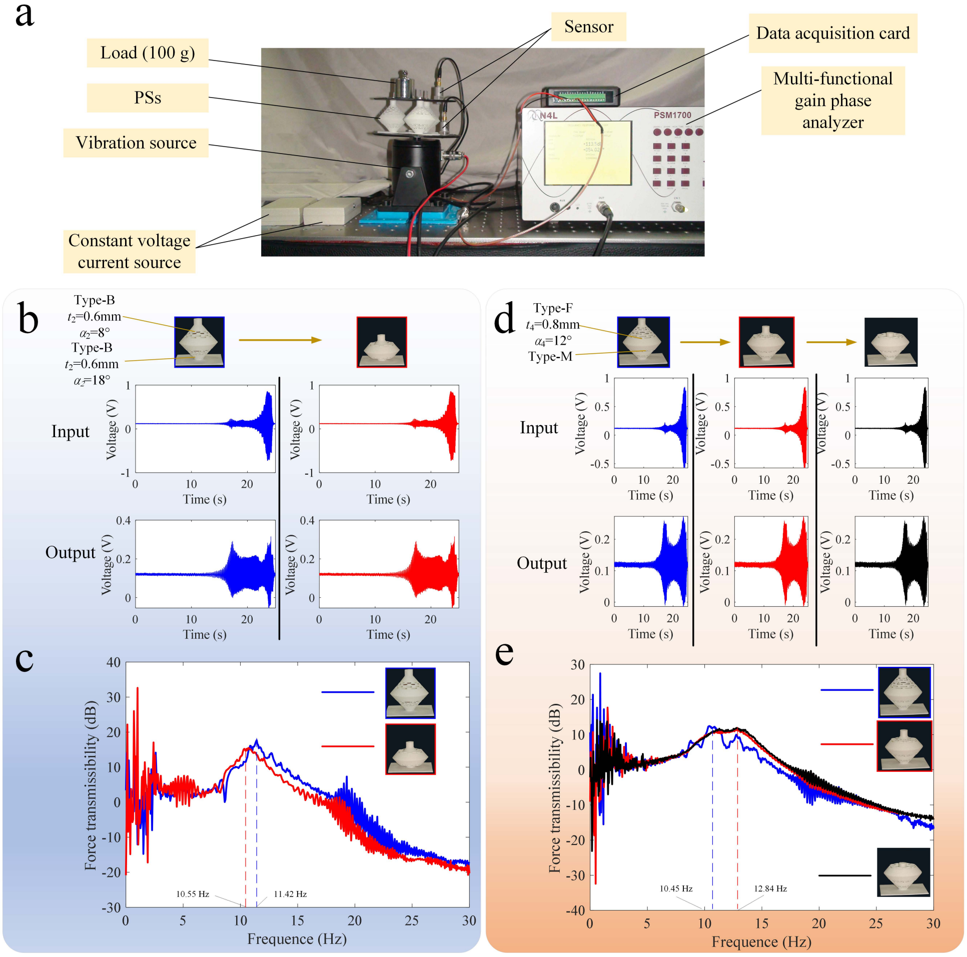

Figure S25 Dynamic information transfer in customized frequency bands. a).

Experimental setup used to measure acceleration transmissibility. b)-c). Force transfer characteristics can be fine-tuned for PS combined time and frequency domain signals. d)-e). PS combinations with adjustable force transmission characteristics and material heights.

Captions for Movies

Movie S1: Loading and unloading of four types of perforated shells.

Movie S2: Demonstration of metamaterials with multi-layer information storage.

Movie S3: Repeatability testing of magnetic drive perforated shell.

Movie S4: Multi-layer information storage under magnetic actuation.

Movie S5: Remote decoding and reading of encapsulated metamaterials.

Movie S6: PSs-based metamaterials are used to encrypt information.

Movie S7: Tunable mechanical logic gates based on perforated shells.

Movie S8: Perforated shells-based metamaterials as an adaptive pressure deliver.

Movie S9: Magnetic decoupling of adjacent unit-cells.

Movie S10: Demonstration of additional states of perforated shells.

Movie S11: Multistable behaviors of PS after 100 cycles of loading.
